# Calcium modified mesoporous silica from marble for the removal of cadmium, lead, chromium, iron, and manganese from Siwa Oasis groundwater

**DOI:** 10.1038/s41598-025-15802-2

**Published:** 2025-08-25

**Authors:** Mohamed Hamdy Eid, Attila Kovács, Péter Szűcs, Mohamed Shaban, A. M. Elbasiony, Ahmed Mehaney, Haifa A. Alqhtani, Ahmed A. Allam, Mostafa R. Abukhadra

**Affiliations:** 1 Institute of Environmental Management, Faculty of Earth Science, University of Miskolc, Miskolc- Egyetemváros, 3515 Hungary; 2 Geology Department, Faculty of Science, Beni-Suef University, Beni-Suef, 65211 Egypt; 3 Department of Physics, Faculty of Science, Islamic University of Madinah, P. O. Box: 170, 42351 Madinah, Saudi Arabia; 4 Department of Chemistry, College of Science, Northern Border University (NBU), Arar, Saudi Arabia; 5 Physics Department, Faculty of Science, Beni-Suef University, Beni Suef, 62512 Egypt; 6 Department of Biology, college of Science, Princess Nourah bint Abdulrahman University, P.O. BOX 84428, 11671 Riyadh, Saudi Arabia; 7 Department of Biology, College of Science, Imam Mohammad Ibn Saud Islamic University, 11623 Riyadh, Saudi Arabia; 8 Geosciences Department, College of Science, United Arab Emirates University, Al Ain, 15551 United Arab Emirates; 9 Applied Science Research Center, Applied Science Private University, Amman, Jordan

**Keywords:** Siwa oasis, Groundwater, Heavy metals, Mesoporous silica, Adsorption, Pollution remediation, Chemical engineering

## Abstract

This study assessed the health risks of heavy metal contamination in groundwater in Siwa Oasis, Egypt’s northwestern desert, and their potential decontamination using a marble-based nanoporous Ca-MCM-41 structure as an adsorbent. Fe, Cd, Cr, Pb, and Mn contents exceeded World Health Organization (WHO) guidelines with potential non-carcinogenic risks and carcinogenic risks based on the hazard index (HI) and Monte Carlo simulations. Ca-MCM-41 showed significant performances in the removal of most of these toxic ions with batch saturation uptake capacities of 239 mg/g Cd(II), 252 mg/g Fe(II), 308 mg/g Pb(II), 132 mg/g Cr(VI), and 154.7 mg/g Mn(II). The batch adsorption behavior display monolayer, homogenous, multi-ionic, nonparallel properties. The adsorption energies (< 8 kJ/mol) highlight the impact of the physical mechanisms and potential regeneration value. The column study using the structure as a fixed bed (3 cm thickness) reflected successful retention for 148.9 mg (Cd (II)), 161.5 mg (Fe (II)), 179.6 mg (Pb (II)), 103.2 mg (Cr (VI)), and 123.7 mg (Mn (II)). The realistic treatment of groundwater in Siwa Oasis demonstrates removal percentages of 84.2% (Cd (II)), 48.8% (Fe (II)), 84.8% (Pb (II)), 52.6% (Cr (VI)), and 52.6% (Mn (II)), considering the variation in the starting concentration and the competitive effect of other pollutants.

**Supplementary Information:**

The online version contains supplementary material available at 10.1038/s41598-025-15802-2.

## Introduction

Chemical pollution of freshwater resources represents a major challenge to both ecological integrity and human well-being, jeopardizing long-term security^1^. The persistent, unregulated release of heavily contaminated effluents from industrial, agricultural, and mining operations further exacerbates water quality deterioration and triggers profound environmental and ecological disruptions^2,3^. In aquatic systems, heavy metals—whether present as free ions or bound in complexes—pose serious threats to ecosystem balance and public health^2,4^. These pollutants are marked by their high toxicity, resistance to biodegradation, carcinogenic potential, and tendency to bioaccumulate in both animal and human tissues^4–6^. Major contributors to this contamination include mining, manufacturing, and nuclear energy industries, which discharge toxic metal ions—such as cadmium, zinc, chromium, mercury, iron, lead, manganese, and barium—into natural waters^7–9^.

Cadmium (Cd(II)) is a highly hazardous metal, and its concentration in drinking water must not exceed 0.003 mg/L, as stipulated by international health and safety guidelines^10^. Exposure to Cd(II) has been linked to a wide range of serious health complications, including pulmonary edema, both acute and chronic illnesses, itai-itai disease, emphysema, liver toxicity, hypertension, reproductive organ damage, kidney impairment, and bone demineralization such as osteomalacia^11,12^. Beyond its effects on human health, Cd(II) also poses significant risks to agriculture, as it disrupts seed germination, stunts plant growth, inhibits root development, and interferes with leaf formation—ultimately undermining crop yield and food security^13^. Elevated levels of Cd (II) also negatively impact aquatic ecosystems, harm marine life, and reduce the economic value of fish stock^3^. Guidelines from both the World Health Organization and the American Water Works Association cap cadmium (Cd(II)) levels in drinking water at 0.005 mg/L^14^. Similarly, lead ions (Pb (II)) are pervasive, highly toxic, non-biodegradable contaminants with chronic neurotoxic effects. These ions frequently enter aquatic environments through various industrial processes and, when present in drinking water, can cause mental disorder, anorexia, and severely affect the human circulatory system, brain function, and reproductive health^15,16^. The United States Environmental Protection Agency (EPA) has established a maximum allowable limit of 0.05 mg/L for lead (Pb(II)) in drinking water to safeguard public health^17,18^.

Hexavalent chromium (Cr (VI)) is highly toxic, highly soluble in water, and poses severe risks to living organisms and the environment^19,20^. This hazardous trace element exists in aqueous systems in various forms, including chromate species (Cr(OH)₂⁺, Cr(OH)₃, and Cr(OH)₄⁻) and dichromate species (Cr₂O₇²⁻, HCrO₄⁻, and CrO₄²⁻)^21,22^. Globally, millions of individuals are impacted annually by water contaminated with chromium, raising significant environmental and public health concerns^23,24^. Anthropogenic activities, such as ore processing, electroplating, and leather tanning, are the primary sources of chromium contamination in groundwater^19,25^. Cr(VI) is a hazardous metallic ion associated with genotoxic, cytotoxic, and phytotoxic effects, and prolonged exposure can lead to serious chronic health issues, including mutagenesis, liver failure, carcinogenesis, central nervous system disorders, skin irritation, anemia, lung cancer, diarrhea, kidney damage, and vomiting^19,21,26^. Regulatory agencies such as the World Health Organization (WHO) and the United States Environmental Protection Agency (USEPA, 2021) have established strict limits for Cr(VI) in water. The maximum permissible concentration of Cr(VI) in inland surface water is 0.1 mg/L, while in drinking water, it must not exceed 0.05 mg/L^27,28^.

Iron and manganese are commonly found in well groundwater due to the geological characteristics of groundwater, particularly the presence of rocky beds^29^. Elevated concentrations of the two metals in drinking water and groundwater wells are associated with significant health and economic challenges^30,31^. While Fe (II) is colorless in its dissolved state, exposure to air oxidizes it to Fe (III), which forms insoluble precipitates. This transformation results in a metallic taste, reddish discoloration, and an unpleasant odor in water^32^. The health impacts of Fe(II) include lung embolism, chronic toxicity, nerve damage, impotence, and bronchitis^33^. Exposure to iron concentrations as high as 1500 mg/L can damage blood tissues in children, while in adults, it can lead to skin conditions, digestive disorders, and dental issues^32^. According to drinking water standards, the maximum allowable concentration of iron is set at 0.3 mg/L, as higher levels are unsuitable for both domestic and industrial applications^29^. Also, manganese is known with its neurotoxic effects, which may result in neurological disorders, degenerative brain conditions, and disruptions in key bodily systems, such as the reproductive, cardiac, and respiratory systems^31,33^. Additionally, its presence in water can cause aesthetic problems, including staining household items and imparting a bitter taste^30,31^. Prolonged exposure to manganese has also been associated with pipe corrosion and health issues, such as bronchitis, hyperactivity, and emotional instability^31,34^. According to the World Health Organization’s guidelines, the acceptable concentrations for manganese and iron in drinking water are 0.3 mg/L and 0.05 mg/L, respectively^31,35^.

Extensive research has demonstrated that methods such as adsorption, flocculation, nanofiltration, biological degradation, membrane separation, ion exchange, and coagulation are effective for the removal and extraction of various metals^36–38^. Adsorption methods, in particular, have attracted considerable interest due to their cost-effectiveness, high efficiency, and potential for regeneration in the removal of metal ions^39–41^. Studies have further highlighted that adsorption using novel nanostructures offers an inexpensive, efficient, reliable, simple, accessible, and environmentally friendly solution for the elimination of a wide range of water contaminants^42,43^. The selection of suitable adsorbent materials is influenced by various factors, including production costs, ease of fabrication, and availability of precursors, adsorption performance, reusability, uptake rate, sustainability, selectivity, safety, and chemical reactivity^44,45^. Consequently, numerous investigations have focused on the development of innovative adsorbents derived from inexpensive and readily available natural minerals^46,47^. Natural materials, such as rocks and minerals, are particularly favored for their economic and environmental benefits^48^. Among these, mesoporous silica nanoparticles have emerged as promising candidates for water purification applications^49,50^. Mesoporous silica materials, such as MCM-41, are highly regarded for their well-ordered nanoporous structures (2–50 nm), small particle sizes, large surface areas, high thermal stability, and excellent adsorption properties^51,52^. Moreover, the presence of active silanol groups within the MCM-41 framework enhances interaction and bonding with a variety of dissolved chemical compounds^53^.

Our previous studies demonstrate significant efficiency for calcium doped MCM-41 which synthesized using different carbonate rocks either as adsorbents or substrates for photocatalysts^48,53^. In this study, calcium-containing mesoporous MCM-41 structure from natural marble as calcium precursor was investigated as efficient adsorbent for the removal of Cd (II), Pb (II), Cr (VI), Fe (II), and Mn (II) ions from aqueous solutions. The research emphasized key practical variables and included equilibrium analysis based on the principles of statistical physics. The theoretical equilibrium analysis considered steric parameters (such as saturating adsorption capacity and occupied active sites) as well as energetic factors (including adsorption energy, internal energy, enthalpy, and entropy). Additionally, the performance of the synthesized material was assessed in a fixed-bed column adsorption system for the five targeted metal ions. This evaluation focused on the column’s mathematical breakthrough parameters and its dynamic adsorption behavior. The study also included the practical application of the material for the removal of Cd (II), Pb (II), Cr (VI), Fe (II), and Mn (II) ions from groundwater samples collected from various wells in the Siwa Oasis.

## Hydrogeological site description

**Fig. 1:**
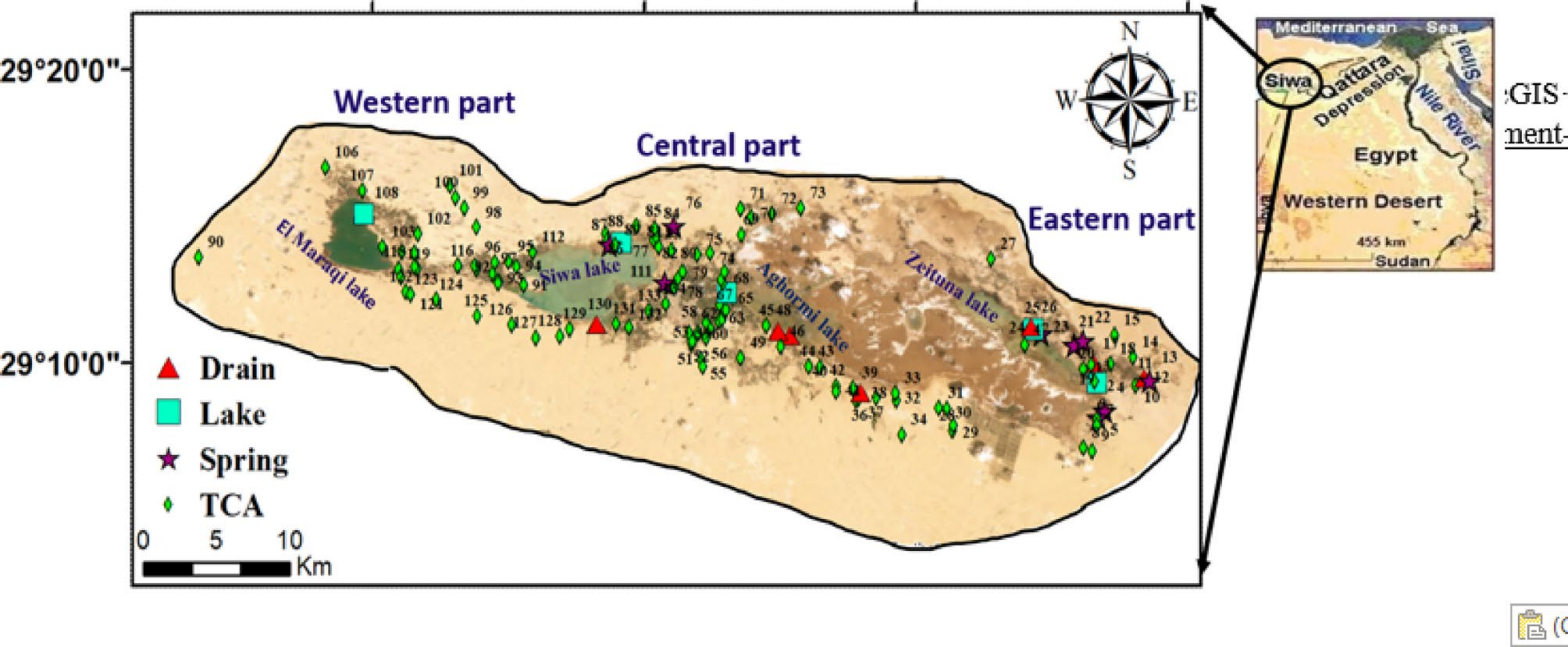
Location map for the studies are and the sampling points sites covering the investigated region (created using ArcGIS Pro 2.8.8 (Esri Inc., 2022; https://support.esri.com/en-us/patches-updates/2022/arcgis-pro-2-8-patch-8-2-8-8-announcement-8074).

Siwa Oasis, situated in Egypt’s northern Western Desert, spans approximately 1100 square kilometers. Located around 320 km south of the Mediterranean Sea, the oasis is home to an estimated 23,000 residents^54^ (Fig. 1). Its economy primarily revolves around agriculture, particularly the cultivation of palm trees, olive oil production, and various fruit and vegetable crops. Additionally, industrial activities such as mineral water bottling and olive oil extraction play a significant role in supporting the local economy^54,55^. The region experiences an arid climate, characterized by a high evaporation rate of 16.8 mm/day during summer, which drops to 5.4 mm/day in winter, and receives minimal rainfall, averaging only 10 mm annually. These harsh climatic conditions, combined with its isolation and limited water resources, present considerable challenges for both residents and businesses^54,56,57^.

The geology of Siwa Oasis comprises various hydrostratigraphic units, including surface deposits such as dunes and salt flats (quaternary deposits) that extend to shallow depths. The Miocene and Eocene formations represent the Tertiary Carbonate Aquifer (TCA), composed of limestone and dolomite intercalated with thin layers of shale and clay. Additionally, older geological layers, including Palaeozoic basement rocks and Mesozoic formations like the Lower Cretaceous Nubian Sandstone and Upper Cretaceous shale, are also present^58^. Water resources in the oasis are primarily derived from two main aquifers: the shallow TCA and the deep Nubian Sandstone Aquifer (NSSA). The TCA, mainly the Miocene aquifer, serves agricultural and domestic needs, while the NSSA is primarily utilized for drinking water and irrigation^59^. Environmental challenges such as soil salinization and waterlogging are evident near salt lakes, including Zeitoun (eastern Siwa), Aghormi, Siwa (central Siwa), and Maraqi (western Siwa), highlighting the need for effective water resource management to ensure sustainability^58–60^. Groundwater flow in the NSSA, as inferred from hydraulic head measurements across 27 wells, predominantly follows a southeast-to-northwest (SE-NW) and southwest-to-northeast (SW-NE) direction. Intensive agricultural activities have led to over-extraction, causing a cone of depression in the central part of the oasis, emphasizing the critical importance of managing groundwater extraction to balance agricultural demand and environmental conservation.

## Experimental work

### Materials

Natural marble rock specimens (metamorphic limestone) were obtained from rock units located in the Eastern Desert of Egypt. The chemicals used for the synthesis of mesoporous calcium silicates included cetyltrimethylammonium bromide (CTAB, 98%), sulfuric acid (98%), ethanol (96%), and sodium silicate, all procured from Sigma-Aldrich, Egypt. Standard solutions of cadmium, chromium, lead, iron, and manganese, each with a concentration of 1000 mg/L, were also supplied by Sigma-Aldrich and utilized to prepare the contaminated aqueous solutions.

### Synthesis of Ca-MCM-41 using natural marble

The MCM-41 nanostructures were prepared using marble (metamorphosed limestone) as the calcium source, following the protocol of^61^. In brief, marble carbonate was milled to a particle size of 50–150 μm. A 100 g portion of this powder was suspended in 100 mL of H₂SO₄ and stirred at 650 rpm for 24 h. The resulting CaSO₄ solution was filtered to remove undissolved solids, then combined with 100 mL of methanol. Subsequently, a sodium silicate solution (20 g in 100 mL H₂O) was introduced into the reactor, and CTAB was added dropwise under vigorous stirring (1000 rpm) at 60 °C for 48 h. The white precipitate was collected by filtration, washed until neutral pH, and dried at 70 °C for 12 h. Finally, to eliminate any remaining surfactant, the material was calcined at 650 °C for 5 h.

The synthesis of Ca-MCM-41 was performed following well-established procedures commonly used for mesoporous silica materials^49^. To verify the reproducibility of the synthesis, three independent batches of Ca-MCM-41 were prepared under identical conditions. The synthesized samples exhibited highly consistent morphological and structural properties based on the XRD and SEM analyses.

### Characterization instruments

The crystalline nature and structural features of the synthesized materials were assessed using X-ray diffraction (XRD) with a PANalytical Empyrean diffractometer. Measurements were performed at high-angle (2θ range: 0°–70°) and low-angle (2θ range: 0°–5°) diffraction settings. Structural changes in functional chemical groups occurring throughout the synthesis procedure were characterized by Fourier-transform infrared spectroscopy (FTIR), employing a Shimadzu FTIR-8400 S spectrometer across a wavenumber range from 400 to 4000 cm⁻¹. Scanning electron microscopy (SEM), using a Gemini Zeiss Ultra 55 microscope, was applied to examine the surface morphology of the materials, with samples pre-coated by a thin gold film to enhance image quality. Additionally, porosity and specific surface area measurements were performed using nitrogen (N₂) adsorption–desorption isotherms obtained on a Beckman Coulter SA3100 analyzer after degassing of samples.

### Environmental assessment and remediation studies

#### Sampling and analysis

In February 2022, a field survey was carried out, during which 123 water samples were gathered (Fig. 1)^54^. These included 103 samples from the Tertiary carbonate aquifer (TCA), eight from springs, and 12 from lakes and drainage systems, all collected in polyethylene containers. During the fieldwork, pH and electrical conductivity (EC) were measured using portable devices calibrated daily. A WTW model LF 538 pH meter was used for pH measurements, while a YSI model 35 conductivity meter was employed for EC readings. The inductively coupled plasma (ICP) was used to measure heavy metal concentrations. Distribution maps of sampling locations and analyzed parameters were created using software such as Surfer 16.6.484 and ArcGIS Pro 2.8.8. The measured levels of metallic elements in the water samples based on the mean values of eight toxic metals were 0.12, 4.8, 1.1, 12.7, 1.7, 0.1, 1.2, and 0.03 mg/L for Cd, Cr, Cu, Fe, Mn, Ni, Pb, and Zn, respectively. These values are arranged in decreasing sequence: Fe > Cu > Cr > Pb > Mn > Ni > Cd > Zn. It is important to highlight that the average levels of Fe, Cd, Cr, Pb, and Mn surpassed the thresholds established by WHO, whereas the remaining metals stayed within the permissible ranges.

#### Human health risk assessment

In our previous research, health risk evaluation was carried out using the methodology endorsed by the United States Environmental Protection Agency (EPA)^54,62^. This framework distinguishes between two main categories of health risk: carcinogenic (CR) and non-carcinogenic (NCR) effects^63^. Carcinogenic risk refers to the likelihood of cancer development following long-term exposure to hazardous substances, whereas non-carcinogenic risk encompasses a range of potential health issues, including genetic mutations and teratogenic outcomes. Heavy metals (HMs) present in drinking water typically enter the human body through ingestion and dermal pathways^64^. Accordingly, this assessment focuses on the potential health risks arising from both oral consumption and skin contact, as quantified by Eqs. 1 and 2.

1 $${\text{C}\text{D}\text{I}}_{\text{o}\text{r}\text{a}\text{l}}=\frac{{\text{C}}_{\text{w}}\times \text{I}\text{R}\times \text{E}\text{F}}{\text{B}\text{W}\times \text{A}\text{T}} \times \text{E}\text{D}$$

2 $${\text{C}\text{D}\text{I}}_{\text{d}\text{e}\text{r}\text{m}\text{a}\text{l}}=\frac{{\text{C}}_{\text{a}\text{v}\text{e}}\times \text{E}\text{T}\times \text{E}\text{F}\times \text{K}\text{p}\times \text{S}\text{A}\times \text{C}\text{F}}{\text{B}\text{W}\times \text{A}\text{T}} \times \text{E}\text{D}$$

In this study, *CDI oral* refers to the average daily dose of contaminants ingested, while *CDI dermal* represents the average daily dose absorbed through the skin. The parameter *Cw* indicates the concentration of heavy metals (HMs) in the water sample (mg/L), *IR* is the daily water intake rate (L/day), *EF* denotes the exposure frequency (days per year), and *ED* is the total exposure duration (years). Other factors include *BW* (body weight in kg), *SA* (exposed skin area), *Kp* (skin permeability coefficient), *CF* (conversion factor), and *ET* (exposure time in hours)^65–67^. A comprehensive list of these exposure parameters is provided in Table S1. The hazard quotient (HQ), reference dose (RfD), health risk index (HI), and carcinogenic risk (CR) for both ingestion and dermal exposure routes are calculated and detailed in Eq. 3 through 6. The parameters applied in estimating these health risk indicators follow the U.S. EPA guidelines and are also outlined in Table S1. Additionally, *ABS* denotes the gastrointestinal absorption factor, and *CSF* stands for the cancer slope factor specific to each heavy metal.

3 $${HQ}_{dermal/oral}=\frac{{\text{C}\text{D}\text{I}}_{\text{d}\text{e}\text{r}\text{m}\text{a}\text{l}}{/\text{C}\text{D}\text{I}}_{\text{o}\text{r}\text{a}\text{l}}}{{\text{R}\text{f}\text{D}}_{\text{d}\text{e}\text{r}\text{m}\text{a}\text{l}}{/\text{R}\text{f}\text{D}}_{\text{o}\text{r}\text{a}\text{l}}}$$

4 $${RfD}_{dermal}={RfD}_{oral}\times ABS$$

5 $$HI=\sum HQ$$

6 $$\text{C}\text{R}=\text{C}\text{D}\text{I} \times \text{C}\text{S}\text{F}$$

#### Batch adsorption experiments

Batch adsorption tests were conducted to assess the effectiveness of synthesized Ca-MCM, derived from marble, in removing Cd(II), Fe(II), Pb(II), Cr(VI), and Mn(II) ions from aqueous solutions. The experiments focused on evaluating the impact of solution pH (ranging from 3 to 7), initial metal ion concentrations (25–300 mg/L), and adsorption contact time (20–560 min). All tests were performed under standardized conditions, including an adsorbent dose of 0.3 g/L, a solution volume of 100 mL, and a constant temperature of 293 K. After reaching equilibrium, Ca-MCM particles were separated from the solutions by filtration, and the residual concentrations of the metal ions were measured. The remaining concentrations of Cd(II), Fe(II), Pb(II), Cr(VI), and Mn(II) were analyzed using inductively coupled plasma mass spectrometry (ICP-MS, Perkin Elmer). Calibration standards for the ICP-MS analysis were sourced from Merck (Germany) and verified against certified references from the National Institute of Standards and Technology (NIST). The adsorption capacity of Ca-MCM was determined using the data obtained, applying Eq. 7, where *Qe* represents the adsorption capacity (mg/g), *Co* is the initial metal ion concentration (mg/L), *Ce* is the equilibrium metal ion concentration (mg/L), *V* is the solution volume (mL), and *m* is the mass of the Ca-MCM adsorbent (mg).

7 $${Q}_{e (mg/g)}=\frac{{(C}_{o}-{C}_{e})V}{m}$$

The adsorption behavior of Cd (II), Fe (II), Pb (II), Cr (VI), and Mn (II) using synthetic coral reef-based Ca-MCM was analyzed through established kinetic models, classical equilibrium approaches, and advanced isotherm models based on the principles of statistical physics (Table S2). The kinetic and equilibrium models were evaluated using nonlinear regression to fit the retention data of the studied heavy metal species. The analysis included standard fitting metrics such as the coefficient of determination (*R*^2^) (Eq. 8) and the Chi-squared statistic (*χ*^2^) (Eq. 9). Additionally, the nonlinear fitting performance of recent equilibrium model equations, along with the adsorption data Cd (II), Fe (II), Pb (II), Cr (VI), and Mn (II), was assessed using the determination coefficient (R^2^) and root mean square error (RMSE) (Eq. 10). In the equations, the parameters *m’*, *p*, *Qi*_*cal*_, and *Qi*_*exp*_ correspond to the adsorption outcomes, factors influencing retention, calculated adsorption capacity, and experimentally determined adsorption capacity, respectively.

8 $${\text{R}}^{2}=1-\frac{\sum ({Q}_{e, exp}-{Q}_{e, cal}{)}^{2}}{\sum ({Q}_{e, exp}-{Q}_{e, mean}{)}^{2}}$$

9 $${{\upchi }}^{2}=\sum \frac{({Q}_{e, exp}-{Q}_{e, cal}{)}^{2}}{{\text{Q}}_{\text{e}, \text{c}\text{a}\text{l}}}$$

10 $$\text{R}\text{M}\text{S}\text{E}=\sqrt{\frac{\sum _{\text{i}=1}^{\text{m}}({\text{Q}\text{i}}_{\text{c}\text{a}\text{l}}-{\text{Q}\text{i}}_{\text{e}\text{x}\text{p}}{)}^{2}}{{\text{m}}^{{\prime }}-\text{p}}}$$

#### Fixed-bed column adsorption studies

The removal performance of Cd(II), Fe(II), Pb(II), Cr(VI), and Mn(II) ions from groundwater was examined using a fixed-bed column system constructed from a borosilicate glass tube (15 cm in length, 2 cm internal diameter) packed with Ca-MCM adsorbent. The Ca-MCM material was securely held in place using layers of polyethylene wool and a plastic mesh to prevent loss of the adsorbent during operation. A peristaltic pump controlled the flow of water through the column, ensuring a consistent rate throughout the experiment. Effluent samples were collected and analyzed every 60 min to monitor the column’s treatment efficiency. The pH of the system was maintained at its original value (pH 6) without adjustment, while bed depths were varied from 1 to 3 cm under a fixed flow rate of 5 mL/min. The effectiveness of the Ca-MCM column in removing heavy metal ions was assessed through several performance indicators, including breakthrough time, saturation time, removal efficiency, and the shape of the breakthrough curves. Breakthrough and saturation points were identified at removal efficiencies of 10% and 95%, respectively. Operational parameters crucial to column performance were calculated, such as the volume of treated water (V_eff_), adsorption capacity of the Ca-MCM bed (C_ad_), total adsorbed metal quantity (Q_total_), total metal input (M_total_), equilibrium adsorption capacity (Q_eq_), and maximum removal efficiency (R%). These metrics were derived using Eq. 11 through 16 as detailed in the study.

11 $${V}_{eff}=Q x {t}_{total}$$

12 $${C}_{ad}={C}_{o}-{C}_{eff}$$

13 $${q}_{total}\left(mg\right)=\frac{QA}{1000}=\frac{Q}{1000}{\int }_{t=0}^{t={t}_{total}}{C}_{ad}dt$$

14 $${M}_{total}\left(mg\right)=\frac{{C}_{o}Q{t}_{total}}{1000}$$

15 $${Q}_{eq}\left(mg/g\right)=\frac{{Q}_{total}}{X}$$

16 $$Total removal .,\% (R.,\%)=\frac{{Q}_{total}}{{M}_{total}}\times 100$$

In these equations, *C*_*o*_ and *C*_*eff*_ represent the initial ion concentrations and the residual concentrations in the effluent, respectively. *Q* denotes the volumetric flow rate (mL/min), *t*_*total*_ corresponds to the total operational time (min), *A* symbol represents the area under the breakthrough curves, and *X* refers to the mass of Ca-MCM in the fixed bed (g). This framework enabled a comprehensive assessment of the column system’s adsorption performance.

All adsorption experiments were conducted in triplicate under identical experimental conditions and variable settings. The results presented in the text and plotted in the figures reflect the mean values. The standard deviation was calculated for all data points and found to be less than 4.7% for the batch adsorption experiments and 6.3% for the fixed-bed column tests, confirming the high reproducibility and consistency of the adsorption performance.

## Results and discussion

### Environmental and health risk assessment

Previous investigations revealed significant contamination of groundwater in Siwa Oasis by heavy metals, raising major public health concerns^54^ (Table 1). Concentrations of Fe, Cu, Cr, Cd, Mn, and Pb frequently exceeded WHO drinking water standards, with Fe showing the highest average level (12.7 mg/L) and Zn the lowest (0.03 mg/L)^54^. These findings reflected widespread water quality deterioration in a region highly reliant on non-renewable groundwater resources for domestic and agricultural use. Health risk assessments indicated that ingestion posed a greater threat than dermal exposure, particularly in children. Hazard quotients (HQs) for Cd, Cr, and Pb exceeded the safe threshold in 76.7–95.5% of samples, with children’s exposure notably higher. Though dermal risks were generally lower, Cd and Cr still presented significant threats to children in up to 50.3% of samples^54^.

**Table 1:** The toxic metals measured from 123 samples reported with lower and upper levels and mean values^54^.

Parameters (mg/L) for metals	Max	Mean	Min	SD
pH	8.7	7.9	6.8	0.3
Zn	0.1	0.03	0.0002	0.024
Pb	2.23	0.3	0.002	0.34
Ni	0.72	0.1	0.0001	0.12
Mn	3.37	0.3	0.0002	0.68
Fe	36.2	2.2	0.003	5.35
Cu	15.6	1.1	0.002	3.004
Cr	12.3	0.6	0.0015	1.63
Cd	0.19	0.04	0.002	0.03

Cumulative hazard indices (HI) confirmed the severity of oral exposure risks, with values reaching up to 142 in adults and 542 in children. Most samples, especially in the western and central Siwa Depression, showed HI values above the acceptable level. Carcinogenic risk (CR) assessments similarly revealed substantial long-term cancer risk from oral exposure, particularly in children, with values far exceeding the acceptable threshold for Cd, Cr, and Pb. Monte Carlo simulations reinforced these findings, identifying ingestion of Cd, Cr, and Pb as the dominant source of health risk. While dermal exposures remained largely within safe limits, oral CR and HQ values for children consistently exceeded safety benchmarks. These findings clearly link groundwater contamination to serious health hazards and underline the urgent need for treatment interventions, continuous monitoring, and regulatory measures. Long-term water protection strategies are vital to safeguard public health and ensure the sustainability of this critical resource.

###  Characterization of the used adsorbent

**Fig. 2:**
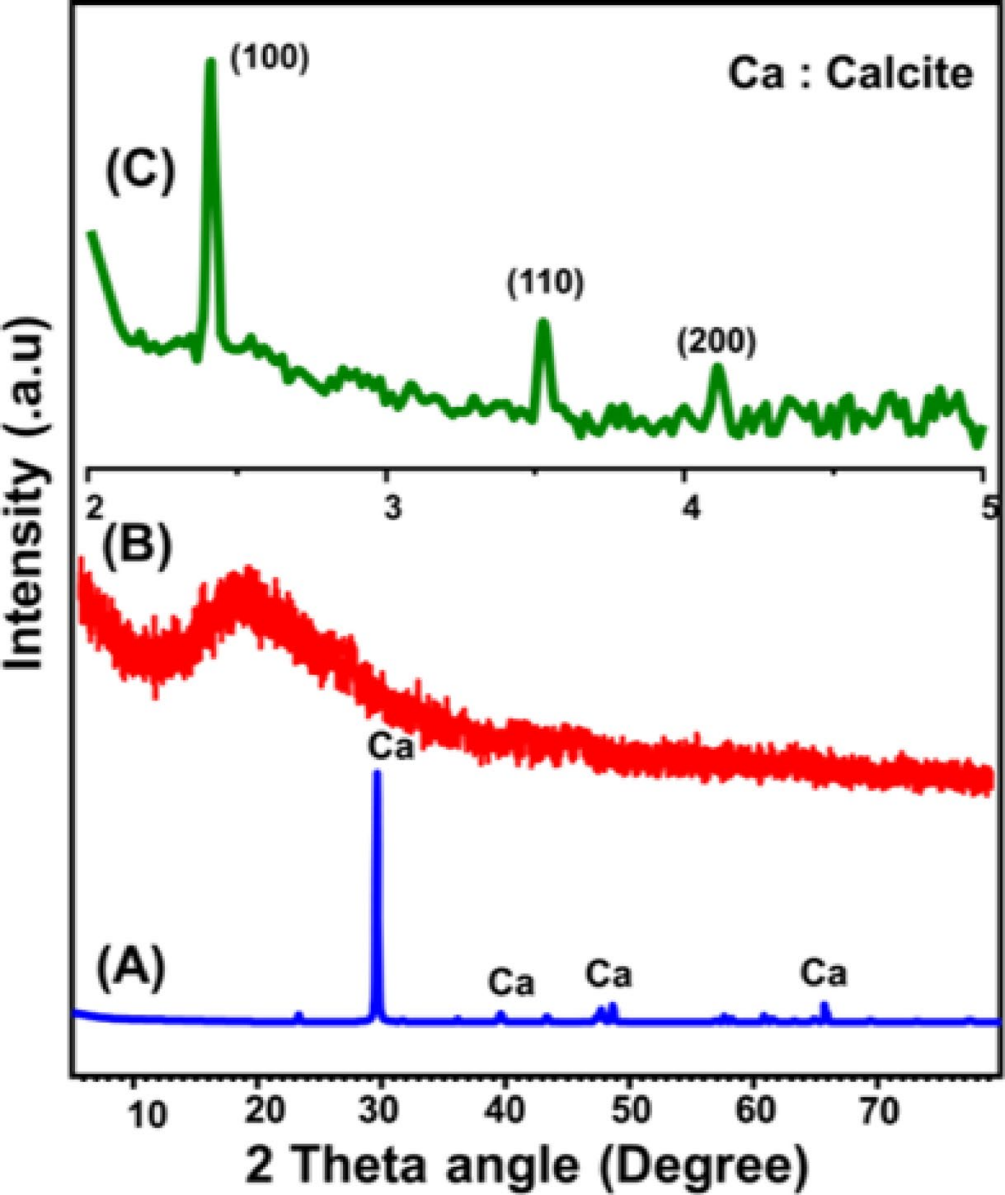
High angle XRD pattern of marble (A) and Ca-MCM structure (B) in addition to the low angle pattern of Ca-MCM (C)^53^.

The structural characteristics of the synthesized materials were examined using both low-angle and high-angle X-ray diffraction (XRD) techniques. High-angle XRD analysis of the metamorphic calcite (marble, M.CA), employed as the calcium source, confirmed its identity as a highly crystalline, pure calcite phase (Fig. 2A)^51^. In the case of the Ca-MCM samples synthesized from marble, the XRD patterns indicated the successful integration of calcium ions into the mesoporous silica-based MCM matrix (Fig. 2B). The high-angle XRD patterns displayed broad reflections centered around 22°, which are typical of the amorphous silica structure comprising the MCM framework^53^. Complementary low-angle XRD measurements revealed the distinctive hexagonal mesostructure of MCM-41 (Fig. 2C), with pronounced diffraction peaks corresponding to the (100), (110), and (200) planes^53^. These features align with the standard reference data from JCPDS 00-049-1712, confirming the preservation of the ordered mesoporous architecture.

**Fig. 3:**
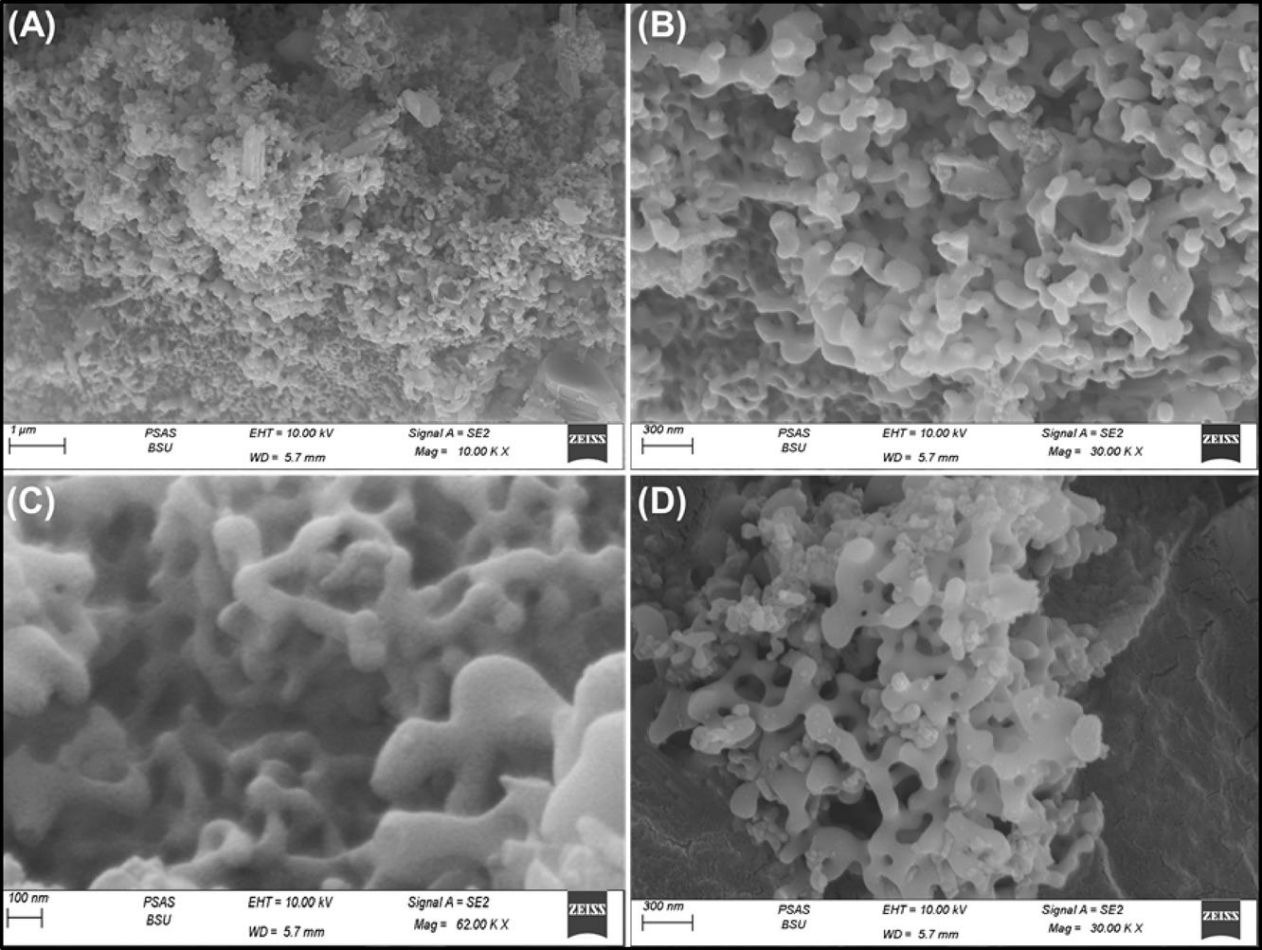
SEM images of the produced Ca-MCM framework using marble as calcium precursor^53^.

**Fig. 4:**
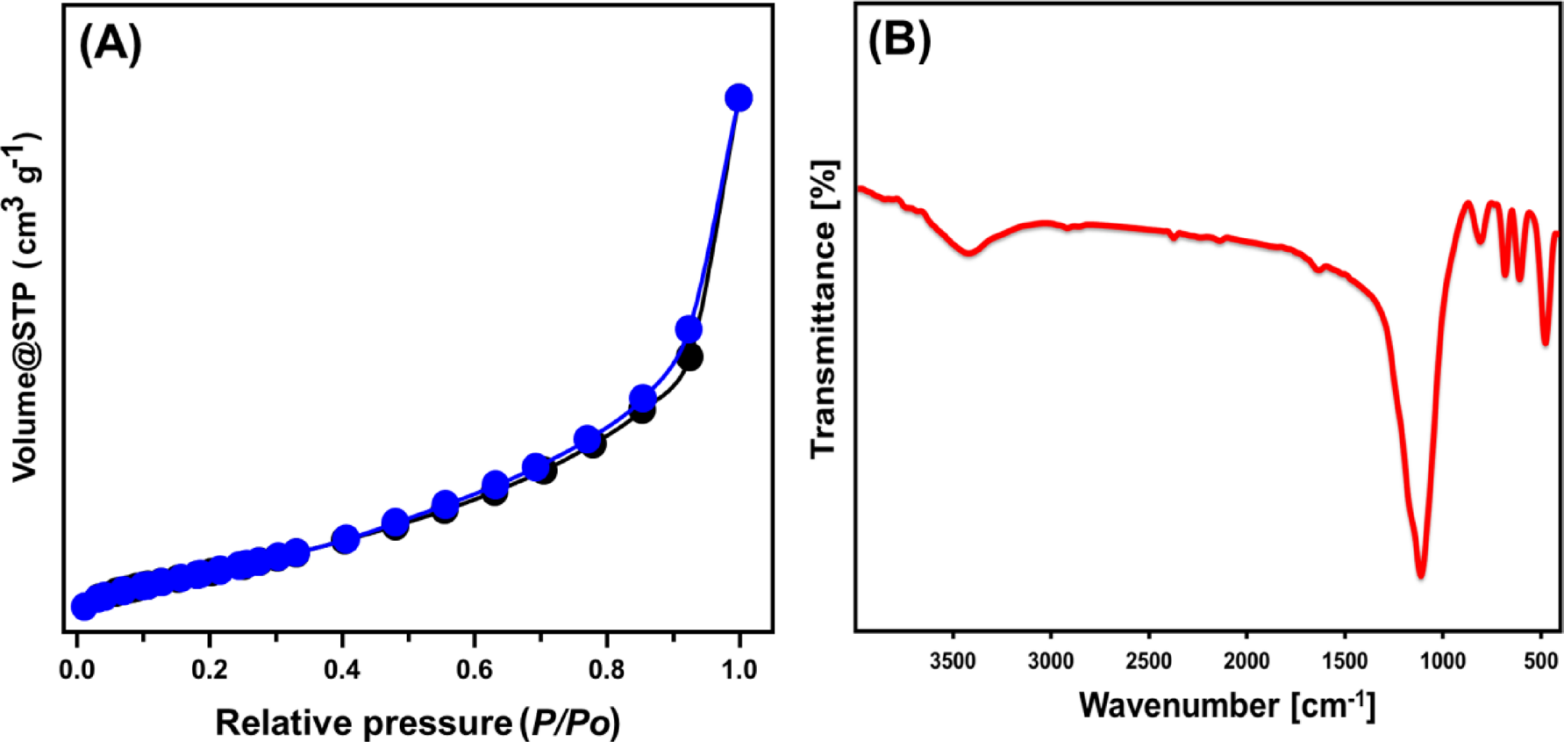
The N_2_ adsorption/desorption isotherm (**A**) and FT-IR spectrum of Ca-MCM framework^53^.

SEM imaging revealed that the synthesized Ca-MCM-41 structure, derived from marble, exhibited a network of intersecting and irregularly shaped nano-grains resembling a worm-like morphology (Fig. 3A and B). This resulted in a highly rugged surface with significant porosity, including both connected and unconnected interstitial nanopores, contributing to enhanced porosity and warping features (Fig. 3C and D). Textural analysis revealed that the synthesized Ca-MCM-41 exhibited a type IV isotherm, based on IUPAC classification, with a steep rise at higher relative pressures due to capillary condensation, confirming the mesoporous nature of the material^68^. Additionally, the isotherm showed an H1 hysteresis loop, typical of mesoporous structures with two-dimensional cylindrical channels (Fig. 4A)^68^. The calculated surface area of the synthesized Ca-MCM-41 was 148.4 m²/g indicating its potential as a promising adsorbent material.

The FT-IR spectrum was analyzed to investigate the chemical structure and functional groups responsible for adsorption activity (Fig. 4B). A strong absorption band around 1100 cm^–1^ was attributed to the asymmetric stretching of Si–O–Si bonds, while lower intensity bands near 800 cm^–1^ and 680 cm^–1^ were assigned to the symmetric stretching of Si–O–Si and O–Si–O bonds, respectively (Fig. 4B)^53,69,70^. The Si–O- bond, a defining feature of the MCM mesoporous structure, was identified by its characteristic band at 470 cm^–1^, and the reactive silanol group (Si–OH) was detected at approximately 3400 cm^–1^ (Fig. 4B)^53,72^. Additionally, the Ca = O band, observed at around 605 cm^–1^, confirmed the incorporation of calcium ions into the marble-based MCM-41 framework (Fig. 4B)^53,72^.

### Adsorption results

#### Batch adsorption studies

##### Effect of pH

**Fig. 5:**
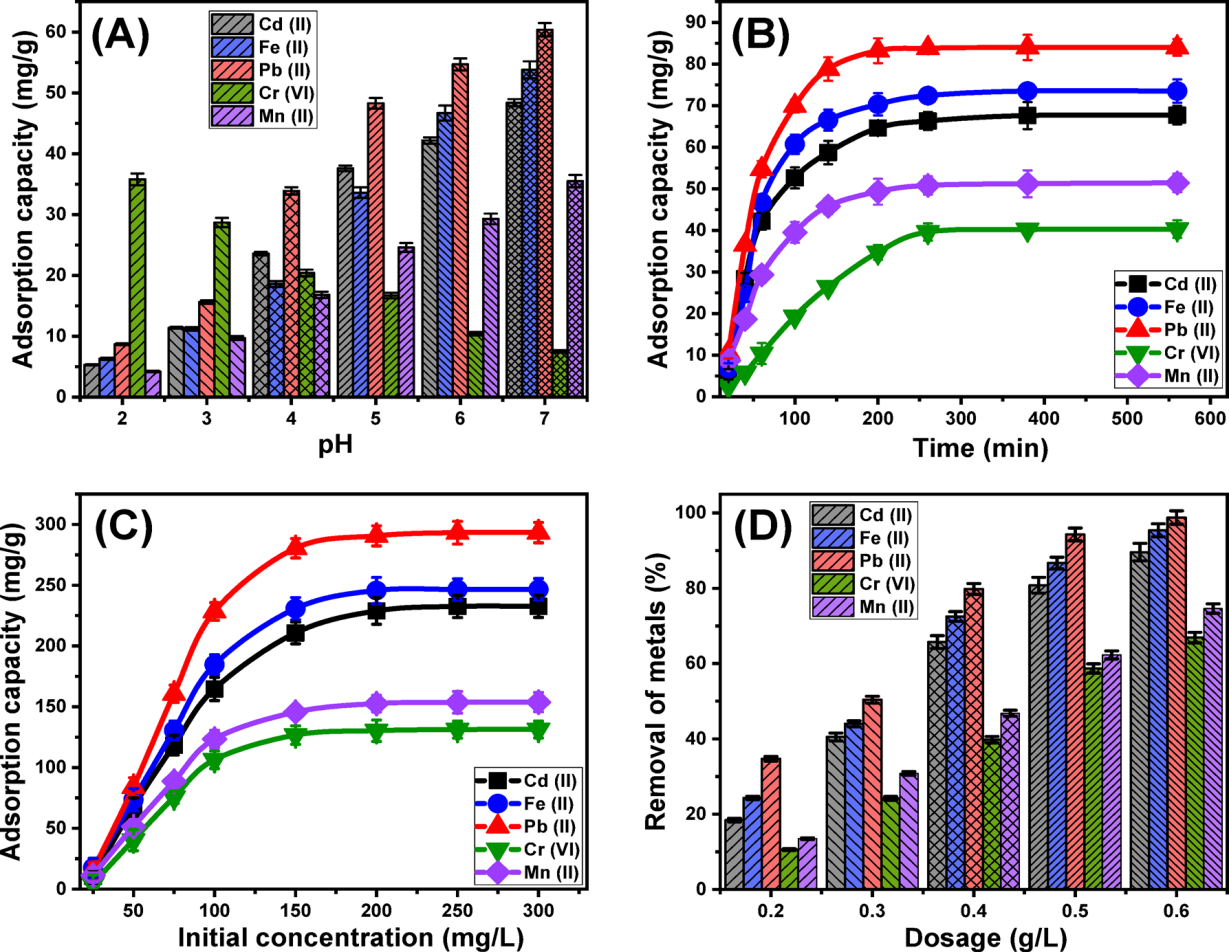
Show the influence of the experimental factors on the removal of the studied five metals by Ca-MCM including (**A**) pH, (**B**) contact time, (**C**) initial concentration, and (**D**) Ca-MCM dosage.

The pH of the aqueous solution plays a critical role in determining the surface charges of the adsorbent and the speciation behavior of dissolved metal ions^73^. In this study, the effect of pH was evaluated across a range of 2 to 7, while maintaining other experimental conditions constant: a contact time of 60 min, temperature of approximately 20 °C, solution volume of 100 mL, metal ion concentration of 50 mg/L, and Ca-MCM dose of 0.3 g/L. The adsorption capacities of Cd(II), Fe(II), Pb(II), and Mn(II) demonstrated a significant increase with rising pH, reaching their maximum at pH 7 (Cd(II) (48.4 mg/g), Fe(II) (53.8 mg/g), Pb(II) (60.4 mg/g), and Mn(II) (35.5 mg/g)) (Fig. 5A). This trend can be attributed to the changes in metal ion speciation and the surface charge properties of Ca-MCM at different pH levels during the adsorption process. Conversely, the adsorption of Cr (VI) exhibited a decrease with increasing pH, from 35.8 mg/g at pH 3 to 7.5 mg/g at pH 7 (Fig. 5A). These findings highlight the ability of Ca-MCM to function as an efficient adsorbent for a variety of metal ions, particularly within the pH range of 6 to 9. This range aligns with the recommendations of the United States Environmental Protection Agency (EPA) for the treatment of industrial wastewater^74^, demonstrating the potential of Ca-MCM for practical applications in wastewater remediation.

The speciation characteristics of dissolved cadmium ions (Cd (II)) indicate that they predominantly exist as free Cd²⁺ ions or in their hydrated state, [Cd(H₂O)₆]²⁺, at pH levels below 8. Beyond pH 8, cadmium transitions into hydroxo complexes, such as [Cd(OH)]⁺^73^. Similarly, lead ions (Pb (II)) are present as Pb²⁺ cations up to a pH of 5. As the pH increases beyond 6, hydroxylated forms of lead, including [Pb(OH)]⁺, [Pb(OH)₂], and [Pb₃(OH)₄]⁺, begin to emerge^18^. For iron ions (Fe (II)), they predominantly exist as Fe²⁺ cations within the studied pH range. However, as the pH exceeds 6.5, Fe (II) oxidizes into Fe (III), which subsequently forms ferric hydroxide precipitates^32^. Manganese ions (Mn (II)) similarly exist as divalent cations (Mn²⁺) across the evaluated pH range, but at pH levels greater than 8, manganese hydroxide begins to precipitate^30,33^. Overall, Cd(II), Fe(II), Pb(II), and Mn(II) maintain positive charges across the examined pH range. At lower pH values, the aqueous solution contains a high concentration of H⁺ ions, which also protonate the reactive groups on the surface of Ca-MCM. The H⁺ ions, due to their greater mobility, outcompete metal ions for the available adsorption sites^30,35^. As the pH increases, the chemical groups on the surface of Ca-MCM gradually deprotonate, resulting in an increased concentration of negatively charged hydroxyl ions on the surface. This reduces competition from H⁺ ions, thereby enhancing the electrostatic attraction and adsorption of positively charged metal ions by MCM-41^30^.

Regarding Cr (VI) ions, their speciation and behavior differ significantly. Under strongly acidic conditions, Cr (VI) primarily exists as Cr₂O₇²⁻, Cr₃O₁₀²⁻, HCrO₄⁻, and Cr₄O₁₃²⁻^73,74^. As the pH increases, these species are gradually replaced by CrO₄²⁻^77^. In highly acidic environments, HCrO₄⁻, which accounts for over 90% of Cr(VI) species, is characterized by a smaller ionic radius. This feature facilitates its dispersion in solution and its immobilization on the surface of Ca-MCM^78^. Consequently, the highly protonated surface of Ca-MCM in acidic conditions enhances electrostatic interactions with Cr (VI) ions, thereby increasing adsorption efficiency^23^. However, as the pH rises, the concentration of OH⁻ ions also increases, leading to reduced surface protonation of Ca-MCM. This results in competition between OH⁻ and Cr (VI) anions for adsorption sites. Furthermore, the negatively charged surface of Ca-MCM repels Cr (VI) anions, reducing the adsorption capacity. The higher affinity of Ca-MCM’s hydroxyl groups for OH⁻ compared to Cr (VI) ions further exacerbates this effect, causing a notable decline in Cr (VI) removal efficiency^75,76^. Therefore, pH 6 was selected to complete all the other tests and value close to the pH values of the investigated real water sample.

##### Effect of contact time

An investigation was carried out to assess the adsorption efficiency of Ca-MCM for the removal of Cd (II), Fe (II), Pb (II), Cr (VI), and Mn (II) ions over a contact time ranging from 20 to 560 min. The study was conducted under controlled parameters, including an initial ion concentration of 50 mg/L, a solution volume of 100 mL, a temperature of 20 °C, a pH of 6, and an adsorbent dosage of 0.3 g/L. The effect of contact time on the adsorption performance of Ca-MCM was specifically evaluated. The results demonstrated a significant improvement in the adsorption of Cd (II), Fe (II), Pb (II), Cr (VI), and Mn (II) ions over time, as indicated by the quantities of ions immobilized and the corresponding uptake rates (Fig. 5B). The adsorption process exhibited a notable increase in efficiency up to approximately 380 min, after which no substantial changes in removal rates or ion immobilization were observed, suggesting the system had reached equilibrium. At equilibrium, the adsorption capacities for Cd (II), Fe (II), Pb (II), Cr (VI), and Mn (II) were determined to be 67.7 mg/g, 73.5 mg/g, 84 mg/g, 40.3 mg/g, and 51.4 mg/g, respectively (Fig. 5B). During the initial phase of the process, adsorption occurred rapidly, likely due to the abundance of reactive and unoccupied adsorption sites on the Ca-MCM nanoparticle surfaces^79^. However, as the contact time increased, the availability of these sites decreased significantly due to the progressive binding of Cd (II), Fe (II), Pb (II), Cr (VI), and Mn (II) ions, resulting in a decline in the adsorption rate. This reduction was attributed to the exhaustion of active functional groups on the Ca-MCM surface, limiting further ion sequestration. In the later stages of the adsorption process, minimal variation or stabilization in adsorption capacity was observed, indicating that equilibrium had been achieved. At this point, the functional sites of Ca-MCM were fully occupied, effectively preventing further binding of ions^37^. These findings highlight the effectiveness and stability of Ca-MCM as an adsorbent for the removal of Cd (II), Fe (II), Pb (II), Cr (VI), and Mn (II) ions under the specified conditions. The equilibrium results further demonstrate its potential for efficient ion removal within the established time frame.

##### Starting concentration

This study evaluated the effect of initial concentrations of Cd (II), Fe (II), Pb (II), Cr (VI), and Mn (II) ions on their maximum adsorption capacities and equilibrium states when using Ca-MCM as an adsorbent. The concentration range of the tested ions varied between 25 and 300 mg/L, while other parameters affecting the adsorption process were kept constant: a solution volume of 100 mL, contact time of 24 h, adsorbent dosage of 0.3 g/L, pH 6, and a temperature of 293 K. The results revealed a positive correlation between increasing initial concentrations of Cd (II), Fe (II), Pb (II), Cr (VI), and Mn (II) ions and enhanced adsorption capacities of Ca-MCM (Fig. 5C). Higher ion concentrations improved diffusion rates, driving forces, and transport dynamics, enabling more interactions between the ions and the active binding sites on the Ca-MCM surface. Consequently, the adsorption efficiencies of Cd (II), Fe (II), Pb (II), Cr (VI), and Mn (II) ions increased significantly with elevated initial ion concentrations^80^. However, this trend persisted only up to a certain concentration threshold. Beyond this point, further increases in the concentrations of Cd (II), Fe (II), Pb (II), Cr (VI), and Mn (II) did not result in improved adsorption performance. This plateau effect is attributed to the saturation of available active sites on the Ca-MCM surface. Determining the equilibrium state was crucial for optimizing the adsorption performance of these ions onto Ca-MCM. The maximum adsorption capacities of Cd (II), Fe (II), Pb (II), Cr (VI), and Mn (II) ions were found to be 232.6 mg/g, 246.5 mg/g, 293.3 mg/g, 131.3 mg/g, and 153.8 mg/g, respectively (Fig. 5C). These results highlight the high efficiency of Ca-MCM as an adsorbent, demonstrating its potential for practical applications in the remediation of groundwater and the treatment of industrial wastewater.

##### Solid dosage

The effect of varying Ca-MCM dosages on the removal efficiency of Cd (II), Fe (II), Pb (II), Cr (VI), and Mn (II) ions was investigated across a range of 0.2 g/L to 0.6 g/L. All other experimental parameters were maintained constant: solution volume (100 mL), contact time (24 h), initial ion concentration (50 mg/L), pH (6), and temperature (293 K). The progressive increase in Ca-MCM dosage and its influence on the removal efficiency of the metal ions is illustrated in Fig. 5D. For all examined metals, a gradual increase in decontamination percentages was observed with higher adsorbent dosages. This improvement can be attributed to the increase in the number of active sites and the exposed surface area of the Ca-MCM particles, enhancing ion adsorption efficiency^34^. However, beyond a dosage of 0.5 g/L, the improvement in removal percentages plateaued, suggesting that the maximum adsorption capacity of Ca-MCM had been reached. Any further increase in the adsorbent mass produced only marginal or negligible effects on the removal efficiency. Experimentally, the removal efficiency of Cd (II) (50 mg/L) by Ca-MCM increased from 18.4% (0.2 g/L) to 40.5% (0.3 g/L), 65.7% (0.4 g/L), 80.8% (0.5 g/L), and 89.9% (0.6 g/L) (Fig. 5D). Similarly, Fe (II) removal improved from 24.3% (0.2 g/L) to 44% (0.3 g/L), 72.5% (0.4 g/L), 86.7% (0.5 g/L), and 95.4% (0.6 g/L). For Pb (II), the removal percentages followed the same trend, increasing from 34.7% (0.2 g/L) to 98.8% (0.6 g/L) (Fig. 5D). The removal efficiency for Cr (VI) rose significantly from 10.6% (0.2 g/L) to 66.9% (0.6 g/L). Similarly, Mn (II) removal increased from 13.5% (0.2 g/L) to 74.6% (0.6 g/L). This highlights the effectiveness of Ca-MCM in the adsorption of the five metals, with optimal performance achieved at higher doses.

##### Kinetic studies

**Fig. 6:**
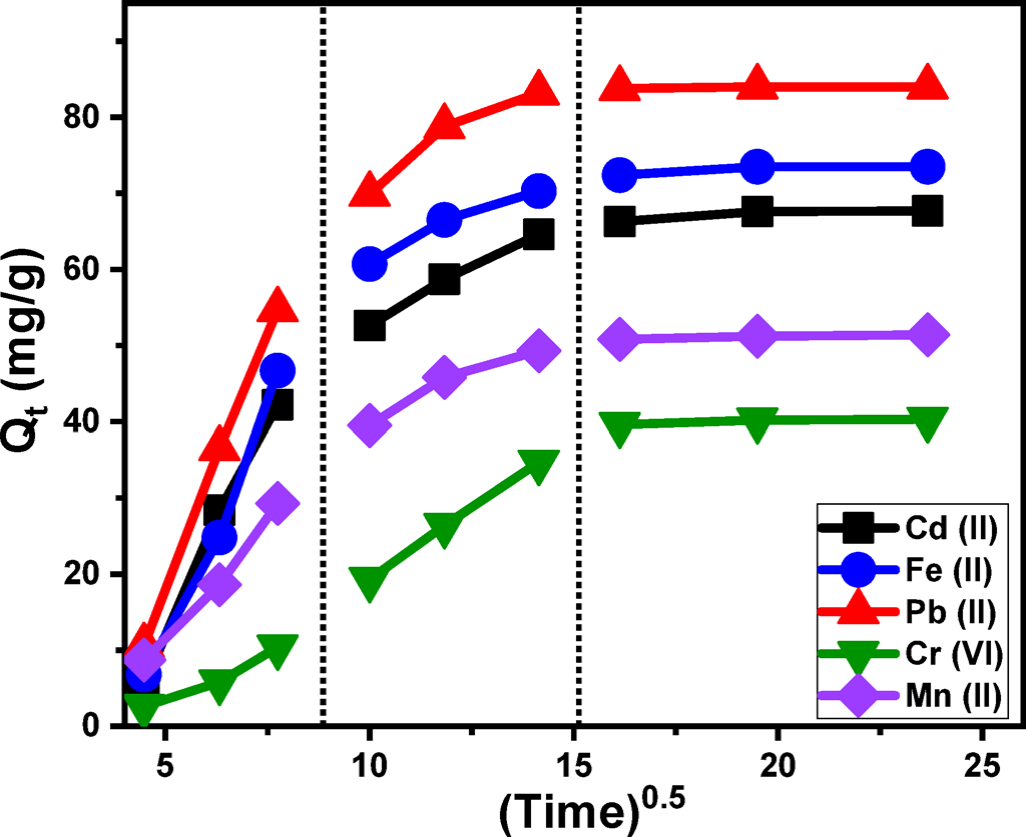
The Intra-particle diffusion behaviors of the five species of heavy metal ions during their adsorption by Ca-MCM.

Intra-Particle diffusion behavior: The analysis of intra-particle diffusion dynamics during the adsorption of Cd (II), Fe (II), Pb (II), Cr (VI), and Mn (II) ions onto Ca-MCM offers critical insights into the underlying adsorption mechanisms and interaction pathways. The adsorption curves presented in Fig. 6 demonstrate three distinct regions with varying slopes, indicative of multiple concurrent adsorption stages and diffusion processes^81^. The adsorption process can be categorized into three main phases:

A. External adsorption (surface interactions):

In the first stage, metal ions interact directly with accessible surface sites on the Ca-MCM framework, where adsorption chiefly takes place on the particle exterior through surface adsorption mechanisms. The efficiency of this phase hinges on the number of available active receptor sites on the Ca-MCM surface, making it vital for initiating metal removal. These surface-level interactions drive the majority of contaminant uptake during this early remediation step (Fig. 6).

B. Internal adsorption (layered adsorption and diffusion):

As external surface sites become saturated, the process transitions into the second phase, characterized by layered adsorption and intra-particle diffusion. The metal ions penetrate deeper into the internal nanoporous structure of Ca-MCM and bind to the internal sites. The diffusion of metal ions to both the surface and interior layers significantly influences this phase. This progression from surface-bound adsorption to internal molecular interactions establishes a secondary removal pathway within the adsorbent structure (Fig. 6)^82,83^.

C. Equilibrium or saturation phase:

The final stage of the adsorption process is defined by the attainment of equilibrium, where all available active sites—both on the surface and within the internal structure of the Ca-MCM adsorbent—are fully occupied. At this point, the adsorption capacity stabilizes, and no additional metal uptake occurs through the primary adsorption mechanisms. Instead, the retention of metal ions is primarily governed by molecular interactions and interionic forces, which maintain the bound species at the adsorption sites. This equilibrium phase represents the complete saturation of the Ca-MCM material and marks the conclusion of the adsorption process (Fig. 6)^4,9^.

The results indicate that external adsorption mechanisms dominate during the initial stages, where metals predominantly bind to the surface sites of the adsorbent. The effectiveness of this phase is strongly influenced by the abundance of active surface sites. Following the saturation of surface binding sites, the adsorption process advances into an internal phase, where metal ions penetrate deeper into the porous matrix of the Ca-MCM adsorbent and interact with interior active sites. As the system approaches equilibrium, all available functional groups become fully engaged, and the adsorption rate stabilizes. During this final stage, molecular interactions and interionic forces contribute significantly to the enhanced retention of metal ions. This stepwise transition—from initial surface attachment to intraparticle diffusion and eventual equilibrium—highlights the intricate and structured nature of the adsorption process for the five investigated metals on Ca-MCM.

Kinetic modeling: A thorough grasp of adsorption kinetics is fundamental to deciphering the underlying physical and chemical processes that control adsorption behavior, particularly the roles of mass transfer and molecular-level interactions that directly affect adsorption performance over time^84^. In this context, the kinetic profiles of Cd(II), Fe(II), Pb(II), Cr(VI), and Mn(II) ions interacting with Ca-MCM were systematically analyzed using two widely recognized kinetic models: the pseudo-first-order (PFO) and pseudo-second-order (PSO) equations. These models offer critical insights into the temporal dynamics of adsorption and assist in elucidating the mechanistic pathways responsible for contaminant removal. The PFO model was applied to describe the rate at which metal ions adhere to available surface sites, which is typically indicative of systems where physical adsorption mechanisms predominate. Conversely, the PSO model was employed to assess kinetic behavior where chemisorption plays a more prominent role, highlighting the contribution of chemical bonding and surface-specific properties of the adsorbent over time^84^. By utilizing both models, a comprehensive analytical framework is established, enabling a deeper understanding of the complex interactions driving the adsorption of metal ions from aqueous solutions.

**Fig. 7:**
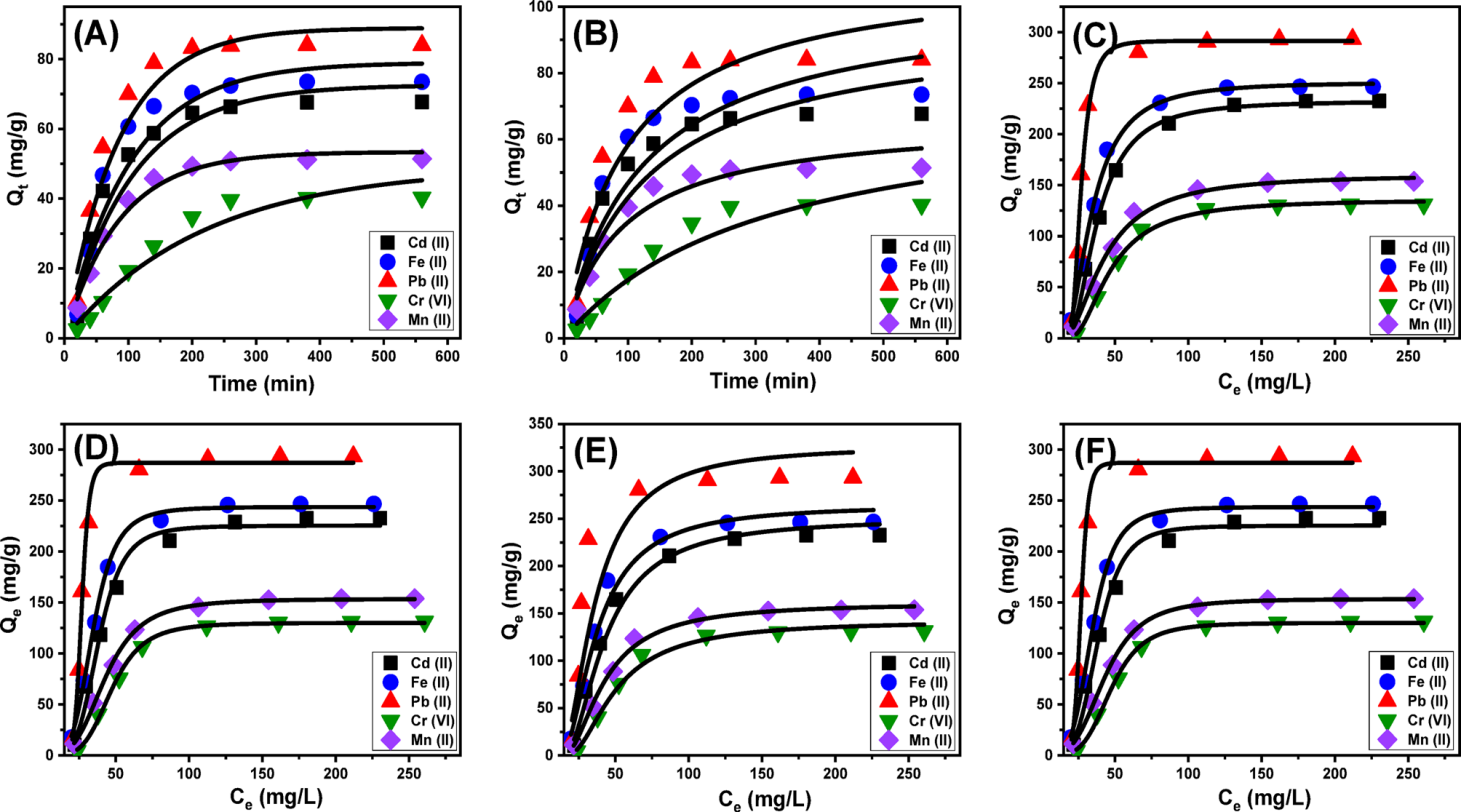
Show fitting of the adsorption behaviors of the studied metals with the different kinetic and isotherm models including (**A**) Pseudo-First order kinetic model, (**B**) Pseudo-Second order kinetic model, (**C**) Langmuir isotherm model, (**D**) Freundlich isotherm model, (**E**) D-R isotherm model, and (**F**) Monolayer model with single energetic site.

To evaluate the suitability of the pseudo-first-order (PFO) and pseudo-second-order (PSO) kinetic models, nonlinear regression analysis was employed. The goodness-of-fit was determined using statistical indicators, specifically the correlation coefficient (R²) and chi-squared (χ²) values. As presented in Table 2; Fig. 7A and B, the PFO model (Fig. 7A) demonstrated a markedly better fit to the experimental data than the PSO model (Fig. 7B), indicating that the adsorption behavior of Cd(II), Fe(II), Pb(II), Cr(VI), and Mn(II) on Ca-MCM is primarily governed by physical adsorption processes. The theoretical adsorption capacities estimated from the PFO model were 72.6 mg/g for Cd(II), 79 mg/g for Fe(II), 88.9 mg/g for Pb(II), 49.1 mg/g for Cr(VI), and 53.4 mg/g for Mn(II), which closely aligned with the experimentally observed values (Table 2). This high level of agreement supports the validity of the PFO model and suggests that non-covalent forces, such as van der Waals attractions and electrostatic interactions, are the dominant mechanisms involved in the adsorption process^85,86^.

While the pseudo-first-order (PFO) model provided a superior fit to the experimental data, the pseudo-second-order (PSO) model also showed a reasonable degree of consistency, suggesting that chemical interactions—such as hydrogen bonding and coordination complex formation—play a supporting role in the overall adsorption mechanism. Nevertheless, these chemical contributions appear to be secondary to the predominant physical forces driving the process. This interpretation is consistent with findings in recent literature, which emphasize that although chemisorption can enhance adsorption efficiency, its influence is generally less significant than that of physical adsorption mechanisms^86^. Furthermore, the results imply the potential occurrence of multilayer adsorption. Initially, metal ions may form a chemically anchored monolayer on the adsorbent surface, which can subsequently promote the physical attachment of additional layers. This sequential layering effect may contribute to increased total adsorption capacity^87^.

##### Equilibrium studies

Classic isotherm models: Equilibrium adsorption tests were performed to reveal the partitioning behavior of dissolved contaminants between the solution and the Ca-MCM adsorbent once concentrations surpassed the equilibrium point. These investigations are critical for delineating both the functional performance and the fundamental principles that govern adsorption processes. Isotherm analyses offer valuable information on: (a) the adsorbent’s preferential binding of specific ions at the solid–liquid interface, (b) the maximum theoretical ion uptake per unit surface area, and (c) the overall adsorption capacity of the material^85,88^. In this study, the equilibrium uptake of Cd(II), Fe(II), Pb(II), Cr(VI), and Mn(II) by Ca-MCM was modeled using the Langmuir (Fig. 7C), Freundlich (Fig. 7D), and Dubinin–Radushkevich (D–R) (Fig. 7E) isotherms. Parameters for each model were obtained via nonlinear regression of the experimental data, and the quality of each fit was assessed by comparing their correlation coefficients (R^2^) and chi-square (χ^2^) statistics, as detailed in Table 2.

**Table 2:** The theoretical parameters of the studied the kinetic models, classic isotherm, and advanced isotherm models.

Model	Parameters	Cd (II)	Fe (II)	Pb (II)	Cr (VI)	Mn (II)
Kinetic models						
Pseudo-first-order	K_***1***_ (min^− 1^)	0.0096	0.0098	0.0119	0.0046	0.0117
	Qe _(Cal)_ (mg/g)	72.6	79	88.9	49.1	53.4
	R^2^	0.93	0.93	0.95	0.96	0.98
	X^2^	2.21	2.03	1.46	0.12	0.23
Pseudo-second-order	k_2_ (g mg^− 1^ min^− 1^)	8.34 × 10^− 5^	8.01 × 10^− 5^	9.71 × 10^− 5^	1.66 × 10^− 4^	4.14 × 10^− 5^
	Qe _(Cal)_ (mg/g)	95.5	103.3	111.8	66.5	74.3
	R^2^	0.90	0.90	0.90	0.94	0.94
	X^2^	2.95	2.87	2.87	0.76	0.91
Classic isotherm models						
Langmuir	Q_max_ (mg/g)	235.4	249.7	297.1	135.1	158.5
	b(L/mg)	3.9 × 10^− 8^	1.4 × 10^− 7^	7.9 × 10^− 8^	3.6 × 10^− 8^	1.5 × 10^− 6^
	RL	0.99	0.99	0.99	0.99	0.99
	R^2^	0.98	0.99	0.99	0.99	0.99
	X^2^	1.47	0.16	0.67	0.24	0.19
Freundlich	1/n	0.88	0.74	0.81	0.91	0.75
	k_F_ (mg/g)	2.47	5.37	4.71	2.84	3.02
	R^2^	0.98	0.97	0.98	0.98	0.98
	X^2^	2.16	2.36	1.15	1.03	0.88
D-R model	β (mol^2^/kJ^2^)	0.0076	0.0065	0.0062	0.0079	0.0074
	Q_m_ (mg/g)	250.3	264.3	326.8	142.0	166.7
	R^2^	0.98	0.99	0.90	0.99	0.99
	X^2^	1.44	1.01	4.12	0.38	0.17
	E (kJ/mol)	8.1	8.7	8.9	7.95	8.2
Advanced isotherm model						
Monolayer model of one energy site	R^2^	0.99	0.99	0.99	0.99	0.99
	X^2^	1.47	0.15	0.67	0.23	0.09
	n	4.67	4.45	10.58	4.44	3.56
	Nm (mg/g)	51.2	56.7	29.12	29.87	43.46
	Q_sat_ (mg/g)	239.1	252.3	308.1	132.6	154.7
	C1/2 (mg/L)	38.5	34.4	26.7	47.4	43.1
	ΔE (kJ/mol)	– 5.2	– 7.6	– 4.4	– 5.9	– 6.8

Nonlinear regression was used to compare the fit of each isotherm model to the experimental adsorption data for all five metals. The Langmuir model demonstrated the closest agreement, as reflected by its highest R² and lowest χ² statistics (Table 2). This superior performance implies that Cd(II), Fe(II), Pb(II), Cr(VI), and Mn(II) ions bind to a uniform array of equivalent sites on the Ca-MCM surface, consistent with Langmuir’s premise of monolayer coverage^86,88^. Moreover, all calculated separation factor (R_L_) values fell below 1, indicating that adsorption under these conditions is thermodynamically favorable^89^. The maximum adsorption capacities (Q_max_) predicted by the Langmuir equation were 235.4 mg/g for Cd(II), 249.7 mg/g for Fe(II), 297.1 mg/g for Pb(II), 135.2 mg/g for Cr(VI), and 158.5 mg/g for Mn(II) (Table 2)^81^. The better agreement of the Langmuir model compared to the Freundlich model can be directly attributed to the physicochemical uniformity of Ca-MCM-41. The ordered hexagonal mesoporous channels might provide energetically equivalent adsorption sites, while the uniformly distributed silanol and Ca²⁺-anchored surface groups promote a finite monolayer adsorption. These structural features align with the fundamental Langmuir assumption of homogeneous surfaces with identical binding sites.

The Dubinin–Radushkevich (D–R) isotherm was also utilized to probe the energetic profile of adsorption. Unlike more traditional isotherm models, the D–R framework focuses on the average adsorption energy (E), which can be used to infer the dominant adsorption mechanism—whether physical, chemical, or a combination thereof^88^. Specifically, E values below 8 kJ/mol indicate primarily physical adsorption; values between 8 and 16 kJ/mol suggest a mixed-mode process incorporating both physical and chemical interactions; and values exceeding 16 kJ/mol point to predominantly chemical adsorption, often associated with stronger bonds such as covalent linkages^90^. In this study, the E values associated with metal ion uptake onto Ca-MCM ranged from 8 to 9 kJ/mol, suggesting that the process follows a dual mechanism. Initially, adsorption is governed by physical forces such as electrostatic interactions, which are subsequently strengthened by chemical contributions, including hydrogen bonding^91^. This synergistic interaction enhances both the stability and efficiency of the adsorption system (Table 2). This combination of mechanisms highlights the efficiency of Ca-MCM in achieving a balance between strong adsorption for pollutant removal and the reversibility required for desorption and adsorbent regeneration. The findings underscore the adaptability and efficiency of Ca-MCM in removing pollutants through adsorption, demonstrating its potential for practical applications.

Advanced isotherm models: To gain deeper insight into the equilibrium behavior of the adsorption process, a statistical physics-based modeling framework was applied. This method allowed for a nuanced interpretation of how water-soluble contaminants interact with the chemically active sites present on the Ca-MCM adsorbent surface. These surface functional groups serve as receptor sites that mediate the binding of metal ions. The model incorporated advanced computational simulations that accounted for both spatial (steric) constraints and energetic heterogeneity, thus offering a comprehensive depiction of the adsorption dynamics. Critical parameters derived from the model included the total number of adsorption sites occupied (Nm), the number of metal ions bound per active site (n), the adsorption energy (ΔE), and the saturation adsorption capacity (Q_sat_) corresponding to full coverage for each of the five studied metals. Model validation was conducted using the Levenberg–Marquardt algorithm in conjunction with multivariable non-linear regression analysis (Fig. 7F; Table 2), supporting the assumptions of a monolayer adsorption model with non-interacting, site-specific binding behavior.

Theoretical analysis of the parameter *n* offered valuable information on the spatial distribution of metal ions on the Ca-MCM adsorbent surface, accounting for both lateral and vertical adsorption orientations. When *n* is less than 1, it indicates a predominantly horizontal alignment of metal ions, implying that each adsorption site binds a limited number of ions. This suggests a multi-docking adsorption mechanism primarily influenced by weak, non-covalent interactions. In contrast, *n* values exceeding 1 reflect a vertical or multilayer stacking of ions at individual sites, pointing to stronger binding affinities between the adsorbed species and the functional groups on the adsorbent. These higher values reveal the capacity of the surface sites to host multiple ions simultaneously, likely through cooperative multi-ionic interactions, thereby improving the overall adsorption performance^4,92^. The adsorption behavior of Ca-MCM exhibited *n* values of 4.67 for Cd (II), 4.45 for Fe (II), 10.58 for Pb (II), 4.44 for Cr (VI), and 3.56 for Mn (II), indicating that single sites can accommodate multiple ions of the respective metals (Table 2). These results suggest that the five metals predominantly adsorb in vertical or non-parallel orientations by multi-ionic interaction processes.

The adsorption capacity per site was found to differ among the metals, influenced by factors such as ionic radius and mobility. Each binding site on Ca-MCM was capable of adsorbing up to 4 ions of Mn (II), 5 ions of Cd (II), Fe (II), and Cr (VI); and 11 ions of Pb (II). These differences reflect the different aggregation tendencies of the metals and their interactions with the Ca-MCM surface and the Pb (II) ions demonstrate the higher aggregation behaviors resulting in strong multi-docking effect. The total number of occupied adsorption sites (*Nm*) for Cd (II), Fe (II), Pb (II), Cr (VI), and Mn (II) were 51.2 mg/g, 56.7 mg/g, 29.1 mg/g, 29.8 mg/g, and 43.5 mg/g, respectively (Table 2). These values align with the observed aggregation properties and experimentally determined adsorption capacities. The saturation uptake capacity (*Q*_*sat*_) was found to depend on the density of occupied adsorption sites (Nm) and/or the number of molecules adsorbed per site (*n*). The estimated *Q*_*sat*_ values for Cd (II), Fe (II), Cr (VI), and Mn (II) were consistent with the calculated Nm values while the uptake capacity of Pb (II) depends mainly on the capacity of each site or the aggregation performance, with *Q*_*sat*_ values of 239 mg/g for Cd(II), 252.3 mg/g for Fe(II), 308.1 mg/g for Pb(II), 132.6 mg/g for Cr(VI), and 154.7 mg/g for Mn(II) (Table 2).

The adsorption energy (ΔE) provided valuable insights into the nature of the interactions driving adsorption, distinguishing between physical and chemical adsorption mechanisms. Physical adsorption typically involves binding energies below 40 kJ/mol, whereas chemical adsorption is associated with higher energies, often exceeding 80 kJ/mol. The *ΔE* values were calculated using Eq. 17, which incorporates the solubility (*S*), the gas constant (R), concentrations at half-saturation (C1/2), and temperature (T)^93^:

17 $$\varDelta E=RT ln\left(\frac{S}{C}\right)$$

The results indicated that the adsorption energies for Cd (II), Fe (II), Pb (II), Cr (VI), and Mn (II) were below 8 kJ/mol (Table 1), consistent with the energy range of physisorption. This suggests that the adsorption process is predominantly driven by physical interactions, including hydrogen bonding (ΔE < 30 kJ/mol), electrostatic attractions (ΔE = 2–50 kJ/mol), van der Waals forces (4–10 kJ/mol), and dipole-dipole interactions (ΔE = 2–29 kJ/mol)^93^. Hydrogen bonding likely arises from interactions between hydroxyl groups on the Ca-MCM surface and electronegative atoms (e.g., oxygen) in the metal ions, facilitated by the interactions between chemical structures of the ions and functional groups on the adsorbent^94,95^. The negative *ΔE* values confirm the exothermic nature of the adsorption process, indicating energetically favorable and spontaneous interactions. Electrostatic forces also contribute significantly to adsorption stability, driven by charge-based attractions between the metal ions and the adsorbent surface. The predominance of physisorption mechanisms has practical advantages for wastewater treatment and pollutant removal, as the relatively weak binding energies facilitate efficient desorption and enable the regeneration of Ca-MCM. This reversibility enhances the material’s reusability and economic viability for large-scale remediation applications.

#### Fixed bed column studies

##### The experimental performance of the Ca-MCM bed

**Fig. 8:**
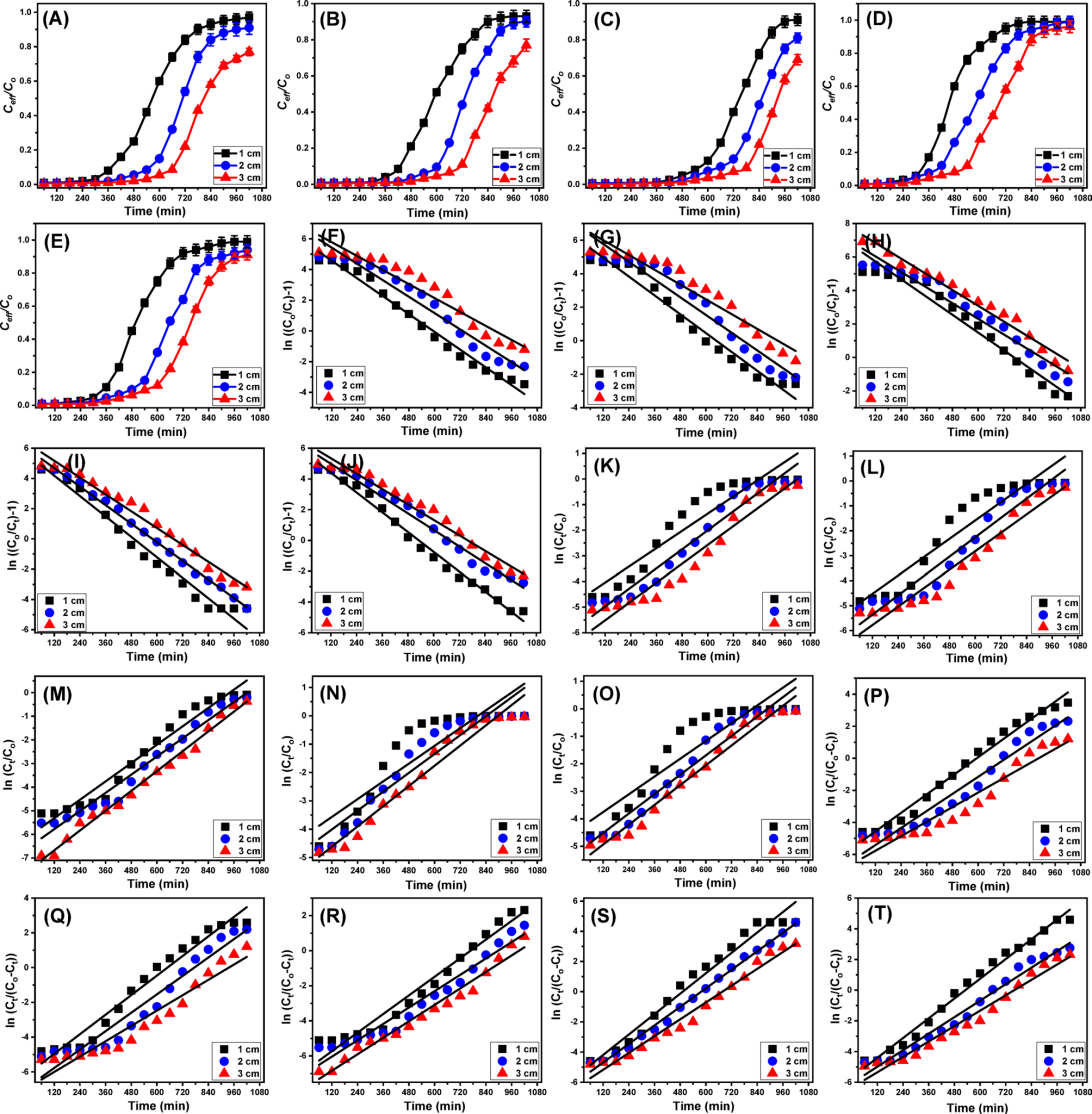
Show the breakthrough curves for the adsorption of studied metals by the Ca-MCM bed (Cd (II) (**A**), Fe (II) (**B**), Pb (II) (**C**), Cr (VI) (**D**), and Mn (II) (**E**)), fitting of the adsorption behaviors with Thomas model (Cd (II) (**F**), Fe (II) (**G**), Pb (II) (**H**), Cr (VI) (**I**), and Mn (II) (**J**)), fitting of the adsorption behaviors with Adams-Bohart model (Cd (II) (**K**), Fe (II) (**L**), Pb (II) (**M**), Cr (VI) (**N**), and Mn (II) (**O**)), and fitting of the adsorption behaviors with Yoon-Nelson Model (Cd (II) (P), Fe (II) (Q), Pb (II) (R), Cr (VI) (S), and Mn (II) (T)).

The effect of Ca-MCM bed thickness on the adsorption performance of column systems designed for the removal of Cd (II), Fe (II), Pb (II), Cr (VI), and Mn (II) was systematically evaluated over a thickness range of 1–3 cm (Fig. 8). The experiments were conducted under controlled conditions: pH 6, an initial metal ion concentration of 5 mg/L, and a flow rate of 5 mL/min for a total duration of 1020 min. Results revealed that increasing the Ca-MCM bed thickness positively influenced both the column’s operational lifetime and its decontamination efficiency for the five metals (Fig. 8; Table 3). For Cd(II) removal, the breakthrough times significantly improved with increased bed thicknesses, reaching 420 min, 600 min, and 720 min for 1 cm, 2 cm, and 3 cm, respectively (Fig. 8A; Table 3). Similarly, the exhaustion times extended to more than 1020 min using the bed at 3 cm thickness. These results correlated with a marked enhancement in Cd (II) removal efficiency, achieving removal percentages of 53.6% (1 cm), 67.5% (2 cm), and 78.4% (3 cm) after treating about 5 L of the polluted water. Therefore, the best results can be obtained using the Ca-MCM bed at 3 cm thickness, the total Cd (II) ions introduced into the column (M_(total)_) were 229.5 mg, with 148.9 mg adsorbed (Q_(total)_). This corresponded to a bed adsorption capacity (C_ad_) of 66.6 mg and a maximum adsorption capacity of 37.7 mg/g (Q_eq_) for the Ca-MCM particles under the experimental conditions (Fig. 8A; Table 3).

Similar trends were observed for the other metals, including Fe (II) (Fig. 8B), Pb (II) (Fig. 8C), Cr (VI) (Fig. 8D), and Mn (II) (Fig. 8E) (Table 3). For a 3 cm Ca-MCM bed thickness, the breakthrough times for Fe (II), Pb (II), Cr (VI), and Mn (II) were 720 min, 800 min, 540 min, and 600 min, respectively, while the corresponding exhaustion times were extended over 1020 min for Fe (II), Pb (II), and Mn (II) and over 960 min for Cr (VI). Removal efficiencies of 82.1% (Fe (II)), 87.1% (Pb (II)), 64.2% (Cr (VI)), and 70.8% (Mn (II)) were achieved after treating approximately 5 L of water. The total metal ions introduced into the column (M_(total)_) were 229.5 mg for the four metals, while the total adsorbed metal ions (Q_(total)_) were 161.5 mg (Fe (II)), 179.6 mg (Pb (II)), 103.2 mg (Cr (VI)), and 123.7 mg (Mn (II)). These corresponded to bed adsorption capacities (C_ad_) of 69.8 mg/g (Fe (II)), 74.1 mg/g (Pb (II)), 54.5 mg/g (Cr (VI)), and 60.2 mg/g (Mn (II)). The maximum adsorption capacities (Q_eq_) for the Ca-MCM particles in the column were determined as 26.9 mg/g (Fe (II)), 29.9 mg/g (Pb (II)), 17.2 mg/g (Cr (VI)), and 20.6 mg/g (Mn (II)) (Table 3). The observed improvements in column performance with increased Ca-MCM bed thickness were attributed primarily to reduced axial dispersion during the mass transfer process, which enhances the diffusion rate of metal ions into the Ca-MCM particles^96^. Additionally, thicker Ca-MCM beds provided extended residence times for the metal ions, ensuring prolonged contact with active adsorption sites, thereby improving metal ion capture efficiency^97^. This synergistic effect of reduced axial dispersion and extended residence times underscores the critical role of bed thickness in enhancing the adsorption performance of column systems for the removal of heavy metals.

However, while greater bed thickness improves contaminant removal, it also increases material consumption, column footprint, and operational costs. In the context of cost-effectiveness and scalability, 3 cm (the highest bed thickness in this study) represents the optimal compromise within the tested range—delivering significant removal efficiency without excessive material usage or pressure drop. For full-scale applications, particularly in resource-limited settings like the Siwa Oasis, this approach minimizes initial investment and operating costs while ensuring reliable contaminant reduction. If stricter compliance with WHO guideline limits is required, incremental bed increases or dual-column configurations can be strategically employed, offering a scalable and economically viable pathway for large municipal systems and decentralized community treatment units.

**Table 3:** The mathematical parameters of the obtained breakthrough curves and the investigated dynamic models.

Mathematical parameters																								
Bed height	Flow rate		Conc.,		C_ad_ (mg/L)		Q (_total_) (mg)			Q_eq_ (mg/g)				M _(total) (mg)_			R.,%	t_b_ (min)			t_s_(min)		V_eff_ (mL)	
Aqueous solutions																								
Cd (II)																								
1 cm	**5 mL/min**		**5 mg/L**		45.6		75.3			37.7				229.5			53.6	420			900		4500	
2 cm	**5 mL/min**		**5 mg/L**		57.4		113.1			28.3				229.5			67.5	600			–		5100	
3 cm	**5 mL/min**		**5 mg/L**		66.6		148.9			24.8				229.5			78.4	720			–		5100	
Fe (II)																								
1 cm	**5 mL/min**		**5 mg/L**		49.3		87.5			43.7				229.5			58.1	480			1020		5100	
2 cm	**5 mL/min**		**5 mg/L**		60.4		123.6			30.9				229.5			71.1	660			–		5100	
3 cm	**5 mL/min**		**5 mg/L**		69.8		161.5			26.9				229.5			82.1	720			–		5100	
Pb (II)																								
1 cm	**5 mL/min**		**5 mg/L**		60.5		124.6			62.3				229.5			71.2	600			–		5100	
2 cm	**5 mL/min**		**5 mg/L**		68.5		156.4			39.1				229.5			80.6	720			–		5100	
3 cm	**5 mL/min**		**5 mg/L**		74.1		179.6			29.9				229.5			87.1	800			–		5100	
Cr (VI)																								
1 cm	**5 mL/min**		**5 mg/L**		36.8		50.3			25.2				229.5			43.3	360			720		3600	
2 cm	**5 mL/min**		**5 mg/L**		45.8		75.8			18.9				229.5			53.9	420			840		4200	
3 cm	**5 mL/min**		**5 mg/L**		54.5		103.2			17.2				229.5			64.2	540			960		4800	
Mn (II)																								
1 cm	**5 mL/min**		**5 mg/L**		40.4		60.1			30.1				229.5			47.6	360			840		4200	
2 cm	**5 mL/min**		**5 mg/L**		53.2		99.4			24.8				229.5			62.6	480			1020		5100	
3 cm	**5 mL/min**		**5 mg/L**		60.2		123.7			20.6				229.5			70.8	600			–		5100	
Real water																								
3 cm	**5 mL/min**		**Cd (II)**		1.78		4.05			0.67				5.51			84.2	780			–		5100	
3 cm	**5 mL/min**		**Fe (II)**		105.4		158.9			26.5				582.9			48.8	420			780		4900	
3 cm	**5 mL/min**		**Pb (II)**		17.3		40.7			6.83				55.1			84.8	840			–		5100	
3 cm	**5 mL/min**		**Cr (VI)**		42.9		69.9			11.6				220.3			52.6	420			840		4200	
3 cm	**5 mL/min**		**Mn (II)**		15.2		24.8			4.13				78.1			52.6	540			960		4800	
Kinetic model																								
Parameters				Thomas Model							Adams–Bohart									Yoon-Nelson model				
Thickness		Flow rate		R^**2**^		K_Th_ ((L/h·mg)		Q_o_ (mg/g)			R^**2**^		K_AB_ (L/g. min)			N_o_ (mg/L)				R^2^		K_YN_ (min^− 1^**)**		τ (min)
Cd (II)																								
1 cm		**5 mL/min**		0.98		0.0019			7.53			0.89	0.00112		6704.9				0.98			0.0096		593.7
2 cm		**5 mL/min**		0.95		0.0018			4.50			0.95	0.00124		3672.5				0.92			0.0088		730.9
3 cm		**5 mL/min**		0.92		0.0015			3.50			0.94	0.00123		2679.8				0.95			0.0076		882.9
Fe (II)																								
1 cm		**5 mL/min**		0.96		0.00187			8.13			0.89	0.00122		6863.9				0.96			0.0094		648.7
2 cm		**5 mL/min**		0.93		0.00176			4.85			0.94	0.00129		3786.2				0.93			0.0088		774.9
3 cm		**5 mL/min**		0.92		0.00147			3.90			0.94	0.00123		2810.9				0.92			0.0074		935.8
Pb (II)																								
1 cm		**5 mL/min**		0.96		0.00176			9.64			0.95	0.00129		7515.8				0.96			0.0088		768.4
2 cm		**5 mL/min**		0.95		0.00155			5.63			0.97	0.00128		4073.6				0.95			0.0077		897.2
3 cm		**5 mL/min**		0.97		0.00156			5.15			0.99	0.00139		2869.3				0.97			0.0078		994.9
Cr (VI)																								
1 cm		**5 mL/min**		0.97		0.00225			6.15			0.80	0.00104		6421.1				0.97			0.0112		491.9
2 cm		**5 mL/min**		0.99		0.00204			3.62			0.90	0.00111		3353.6				0.99			0.0102		578.2
3 cm		**5 mL/min**		0.97		0.00185			2.83			0.96	0.00118		2379.1				0.97			0.0093		677.3
Mn (II)																								
1 cm		**5 mL/min**		0.98		0.00214			6.65			0.84	0.00107		6548.9				0.98			0.0107		530.9
2 cm		**5 mL/min**		0.97		0.00179			4.23			0.94	0.00117		3551.3				0.97			0.0089		676.5
3 cm		**5 mL/min**		0.96		0.00167			317			0.97	0.0012		2501.9				0.97			0.0083		759.7

##### Dynamic modelling of the column

This study utilized three kinetic models—Thomas, Adams-Bohart, and Yoon-Nelson—to characterize the dynamic behavior and adsorption performance of the Ca-MCM-based column system. These models were applied to predict the breakthrough behavior and evaluate the adsorption efficiency of the fixed bed. Specifically, the Adams-Bohart and Yoon-Nelson models were employed to estimate the breakthrough characteristics of the column and its affinity for dissolved pollutants in groundwater, particularly during the initial stages of the decontamination process^96,98^. The parameters derived from these kinetic models provided valuable insights into the actual performance of the column. Key outputs included the saturation concentration, the adsorption capacity of the bed, and the time required to reach 50% breakthrough^97^. The kinetic properties of the Ca-MCM bed were assessed through nonlinear regression analysis using the equations corresponding to the Thomas, Adams-Bohart, and Yoon-Nelson models (Eqs. 18, 19, and 20, respectively). These models are critical for understanding the adsorption mechanisms and optimizing the column’s operational efficiency.

18 $$\text{ln}(\frac{{C}_{o}}{{C}_{t}}-1)=\frac{{K}_{Th{q}_{0}M}}{F}-{K}_{Th}{C}_{o}t$$

19 $$\text{ln}(\frac{{C}_{t}}{{C}_{o}})={K}_{AB}{C}_{o}t-{K}_{AB}{N}_{o}\frac{Z}{{U}_{o}}$$

20 $$\text{ln}\left(\frac{{C}_{t}}{{C}_{o}{-C}_{t}}\right)={K}_{YN}t-\tau {K}_{YN}$$

Thomas model: The Thomas model assumes that the retention of the five metal ions (Cd (II), Fe (II), Pb (II), Cr (VI), and Mn (II)) within the Ca-MCM bed follows second-order adsorption kinetics. According to this model, the column system exhibits reversible adsorption reactions with Langmuir equilibrium characteristics, disregarding the effects of axial dispersion and internal or external diffusion limitations of the metal ions^99,100^. The determination coefficient (*R²*) values obtained across varying Ca-MCM bed thicknesses indicate strong conformity between the adsorption reactions occurring within the fixed bed and the assumptions of the Thomas model (Fig. 10I to L; Table 3). The results reveal a decrease in the equilibrium adsorption capacities of the Ca-MCM as the bed thickness increases from 1 to 3 cm for all five metals.

Adams-Bohart model: The Adams-Bohart model provides valuable insights into the transport behavior of the metal ions within the Ca-MCM fixed bed. This model assumes constant adsorption capacities for the metal ions (Cd (II), Fe (II), Pb (II), Cr (VI), and Mn (II)) during adsorption processes that follow a stepwise isotherm mechanism^100^. The breakthrough data show a strong agreement with the Adams-Bohart model under varying Ca-MCM bed thicknesses and flow rates (Fig. 10M to P; Table 3). According to the model’s predictions, the saturation concentration (*N*_o_) of the five metals decreases consistently as the bed thickness increases (Table 3). These findings demonstrate the relevance of the Adams-Bohart model in describing the adsorption behavior.

Yoon-Nelson model: The Yoon-Nelson model is primarily applied to estimate the saturation time of the Ca-MCM bed during the removal of the five metal ions and to characterize the adsorption kinetics under specific experimental conditions^97^. The adsorption kinetics for column systems following the Yoon-Nelson model are governed by the breakthrough behavior of the Ca-MCM fixed bed, which influences the uptake rates of the metals^96^. High determination coefficient (*R²*) values confirm the consistency of the experimental data with the Yoon-Nelson model (Fig. 10Q to T; Table 3). The model predicts that a Ca-MCM bed with a 3 cm thickness reaches 50% of its saturation capacity after 882.9 min for Cd (II), 935.8 min for Fe (II), 994.9 min for Pb (II), 677.3 min for Cr (VI), and 759.7 min for Mn (II). These results highlight the potential enhancement in column lifetime when using a thicker Ca-MCM bed (Table 3). Optimizing the bed thickness and other operating parameters can lead to significant improvements in the decontamination efficiency and processing capacity of the investigated metal ions over large solution volumes.

## Realistic remediation of groundwater in Siwa Oasis and its environmental–health significance

The remediation potential of CaMCM-41 was validated under realistic conditions using groundwater collected from a representative well in the Siwa Oasis, a desert region where natural geological formations and anthropogenic activities have resulted in elevated concentrations of toxic heavy metals. The initial metal concentrations—Cd(II) (0.12 mg/L), Pb(II) (1.20 mg/L), Cr(VI) (4.80 mg/L), Fe(II) (12.7 mg/L), and Mn(II) (1.70 mg/L)—were all substantially above the World Health Organization (WHO) and USEPA guideline values for safe drinking water. Chronic exposure to these levels presents severe health risks, including neurotoxicity and kidney damage from Pb(II) and Cd(II), carcinogenic effects from Cr(VI), and cumulative toxicity from excessive Fe(II) and Mn(II).

**Fig. 9:**
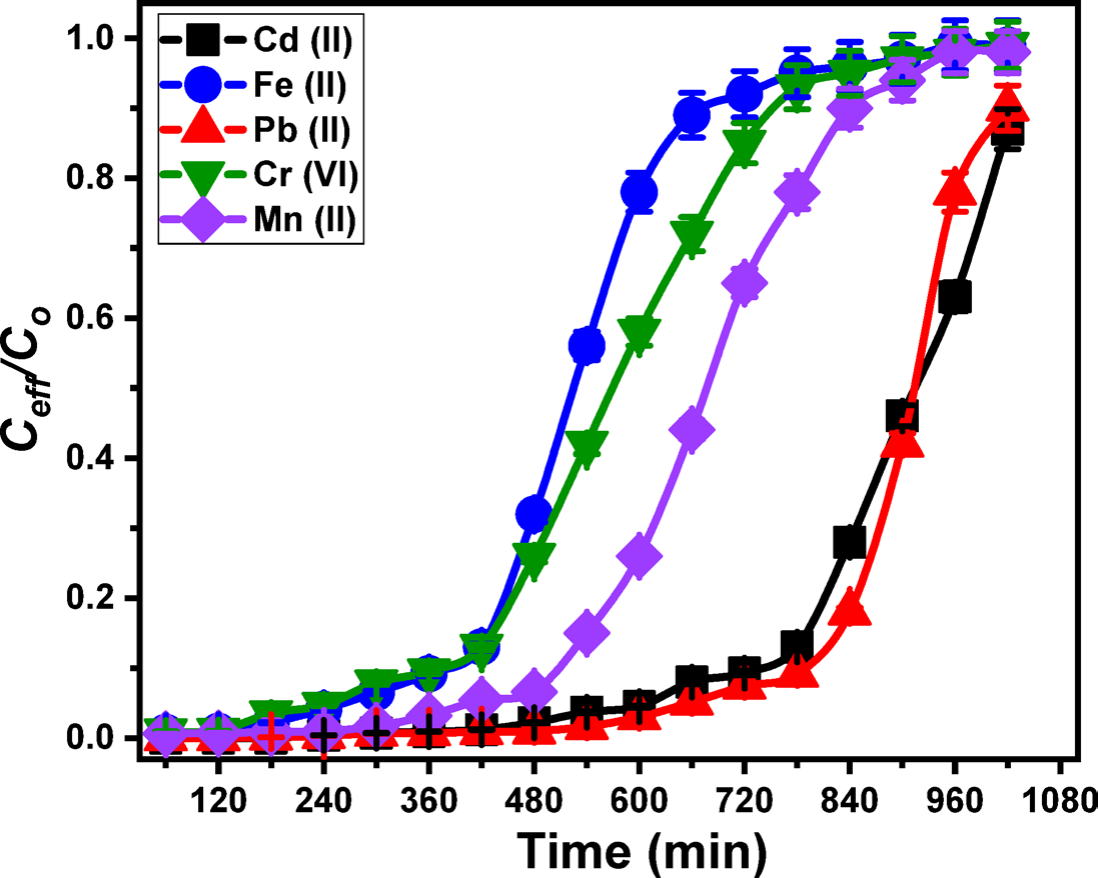
Show the breakthrough curves for the real adsorption of studied metals from the groundwater sample by the Ca-MCM bed.

To simulate realistic treatment conditions, a fixed-bed column packed with a 3 cm layer of CaMCM-41 was operated at an optimized flow rate of 5 mL/min and pH 6.0, for a total flow time of 840 min (Fig. 9; Table 3). Even under the complexity of real groundwater—where competing ions such as carbonate, phosphate, and nitrate coexist and high hardness levels reduce adsorption efficiency—the CaMCM-41 column achieved notable breakthrough and exhaustion times: 780 min and > 1020 min for Cd (II), 420 min and > 780 min for Fe (II), 840 min and > 1020 min for Pb(II), 420 min and > 840 min for Cr(VI), and 540 min and > 960 min for Mn(II) (Fig. 9; Table 3). After processing approximately 5 L of groundwater, the overall removal efficiencies reached 84.2% for Cd (II), 84.8% for Pb(II), 52.6% for Cr(VI), 48.8% for Fe(II), and 52.6% for Mn(II) (Fig. 9; Table 3). Consequently, the post-treatment residual metal concentrations were reduced to 0.016 mg/L for Cd (II), 0.18 mg/L for Pb(II), 2.3 mg/L for Cr(VI), 6.5 mg/L for Fe(II), and 0.8 mg/L for Mn(II). These results highlight a strong selectivity toward Cd (II) and Pb(II), which are the most toxic and health-critical contaminants in this water matrix.

Before treatment, the hazard quotient (HQ) values for Cd(II) and Pb(II) in the raw groundwater far exceeded unity (> 1), indicating an unacceptable noncarcinogenic risk for local populations consuming this water. Cr(VI), recognized as a Group 1 human carcinogen, posed additional long-term cancer risks even at trace levels. Furthermore, the combined hazard index (HI) from all five metals reflected significant cumulative toxicity risks, which directly threaten both human health and aquatic ecosystems dependent on the oasis water sources.

After treatment with CaMCM-41, the substantial reduction in Cd(II) and Pb(II) concentrations to 0.016 mg/L and 0.18 mg/L, respectively, markedly lowered their HQ values, reducing them close to or below the WHO/USEPA thresholds. Although Fe(II), Mn(II), and Cr(VI) levels remained moderately above strict drinking water criteria, the overall decline—reducing Fe(II) by ~ 50%, Cr(VI) by > 50%, and Mn(II) by ~ 53%—significantly decreased the cumulative HI and carcinogenic risk indices. Importantly, the removal of Pb(II) and Cd(II), which contribute disproportionately to both acute and chronic toxicity, offers the greatest health risk mitigation benefit. From an environmental perspective, lowering toxic metal levels in groundwater also protects aquatic life and the soil–water interface ecosystems of the Siwa Oasis. By preventing metal bioaccumulation in aquatic organisms and reducing their transfer along the food chain, this treatment indirectly safeguards both biodiversity and human populations reliant on oasis water for agriculture, drinking, and domestic use.

In summary, the integration of adsorption efficiency with health risk reduction demonstrates that CaMCM-41 is not only a high-performing adsorbent in laboratory tests but also a realistic and sustainable solution for environmental remediation. It directly addresses the pressing public health challenges posed by heavy metal contamination, improves the ecological integrity of oasis water systems, and aligns with global sustainability goals for safe water access in vulnerable regions. Notably, the post-treatment residual concentrations for Cd(II) (0.016 mg/L) and Pb(II) (0.18 mg/L) approached the WHO/USEPA drinking water guideline limits of 0.003 mg/L and 0.01 mg/L, respectively. Mn(II) (0.80 mg/L) and Cr(VI) (2.3 mg/L) also showed significant reductions toward the recommended thresholds (0.4 mg/L for Mn and 0.05 mg/L for Cr(VI)), while Fe(II) decreased from 12.7 mg/L to 6.5 mg/L, approaching the USEPA secondary standard of 0.3 mg/L. While Fe(II) and Cr(VI) require extended treatment (e.g., thicker beds or dual-column systems) for full compliance, the substantial removal of Pb(II) and Cd(II)—the most critical for human health—already represents a significant improvement in water safety and overall risk reduction.

## Comparative and practical significance of ca‑mcm‑41

The development of Ca‑MCM‑41 from natural marble offers a unique convergence of environmental sustainability, technical efficiency, economic feasibility, and industrial scalability that is rarely achieved in a single material. While mesoporous silica such as tetraethyl orthosilicate (TEOS) derived MCM‑41 and SBA‑15 have long been recognized for their high surface areas and well-ordered porosity, their broad application in water treatment remains limited by the cost of high-purity precursors, solvent-intensive synthesis procedures, and the difficulty of scaling laboratory protocols to industrial processes^61,102^.

Marble‑derived Ca‑MCM‑41 directly addresses these limitations. Environmentally, this material transforms marble waste—a widely available byproduct of mining and construction—into a high-value adsorbent, reducing solid waste accumulation and alleviating the environmental burden associated with quarrying and disposal. By substituting virgin silica extraction with waste valorization, the ecological footprint of silica production is markedly reduced. Moreover, the synthesis route is solvent-free and energy-moderate, minimizing greenhouse gas emissions and aligning with sustainable materials engineering principles. Technically, Ca‑MCM‑41 maintains the mesostructural advantages of conventional MCM‑41, including a large surface area and highly ordered pore channels that enhance accessibility and diffusion for contaminant ions. The introduction of calcium ions during synthesis further improves the material’s affinity for divalent and hexavalent metal species, strengthening electrostatic adsorption and ion-exchange mechanisms^61^. This feature enables simultaneous removal of multiple pollutants—Cd(II), Pb(II), Cr(VI), Fe(II), and Mn(II)—which is highly relevant for real groundwater conditions where contaminants coexist. Notably, the material achieved adsorption capacities of 239 mg/g for Cd(II), 308 mg/g for Pb(II), 132 mg/g for Cr(VI), 252 mg/g for Fe(II), and 154 mg/g for Mn(II), values that match or surpass the best-performing adsorbents reported in the literature^103,104^.

From a sustainability perspective, the life cycle of Ca‑MCM‑41 demonstrates clear advantages. It originates from a benign mineral waste, requires no toxic organic reagents, and can be regenerated for repeated use, extending its functional lifetime and reducing post-treatment disposal concerns. This approach resonates with global strategies aimed at integrating waste-derived materials into pollution control technologies, making it not only an environmentally responsible choice but also a model for circular economy practices. Financially, marble-derived Ca‑MCM‑41 provides a highly competitive alternative. Raw marble waste has negligible cost compared to high-purity silica sources like TEOS. In combination with a mild synthesis protocol that avoids specialized reactors and strict atmospheric control, the overall production cost is reduced by 40–60% relative to traditional mesoporous silica. This cost advantage makes it feasible for municipal-scale deployment in resource-limited regions, such as Siwa Oasis, where cost constraints often impede the adoption of advanced materials.

Finally, scalability is one of the most critical and underreported barriers in translating novel adsorbents from laboratory to industrial application. The synthesis of Ca‑MCM‑41, based on simple acid dissolution, surfactant templating, and moderate-temperature calcination, is inherently scalable. It does not depend on costly or chemically intensive reagents or tightly controlled synthesis conditions, enabling both centralized industrial production and decentralized community-scale fabrication. This scalability contrasts sharply with many emerging nanomaterials that remain confined to experimental use due to complex and cost-prohibitive production routes.

## Conclusions

- The findings of this study reveal significant health risks posed by toxic metals contamination in Siwa Oasis, particularly for children, who are more vulnerable to both non-carcinogenic and carcinogenic effects. The elevated levels of Cd (II), Fe (II), Pb (II), Cr (VI), and Mn (II) in water resources exceeding WHO guidelines underscore the need for immediate action to mitigate exposure and protect public health.
- Calcium-modified MCM-41 nanoporous structure was synthesized using natural marble rocks and characterized as a potential adsorbent for Cd (II), Fe (II), Pb (II), Cr (VI), and Mn (II) metal ions with enhanced efficiency, safety, and suitability for practical applications. The structure displays significant performance either in the batch uptake of these ions or the fixed bed column investigation. The batch studies demonstrate saturation uptake capacities of 239 mg/g Cd (II), 252 mg/g Fe (II), 308 mg/g Pb (II), 132 mg/g Cr (VI), and 154.7 mg/g Mn (II). The experimental uptake behaviors were illustrated based on the kinetic, classic, and advanced isotherm modeling. The recognized parameters declare the physical uptake of these ions in homogenous and monolayer forms involved multi-ionic reactions. This implies the facile regeneration of the MCM particles as adsorbents as well as the re-extraction of the sequestrated ions.
- The fixed bed column investigation declared significant efficiency of the Ca-MCM-41 particles for the practical retention of the dissolved ions of toxic heavy metals. The Ca-MCM bed (3 cm) displays total retention capacities for 148.9 mg (Cd (II)), 161.5 mg (Fe (II)), 179.6 mg (Pb (II)), 103.2 mg (Cr (VI)), and 123.7 mg (Mn (II)) from the total pumped metals into the bed (229.5 mg). These values correspond to removal percentages of 78.4% (Cd (II)), 82.1% (Fe (II)), 87.1% (Pb (II)), 64.2% (Cr (VI)), and 70.8% (Mn (II)) from the starting concentration of the feed. The dynamic properties of the bed were illustrated based on the assumptions and parameters of the Thomas, Adams-Bohart, and Yoon-Nelson models.
- The structure was applied effectively in the decontamination of the five metal ions from the groundwater wells in Siwa Oasis. The Ca-MCM bed reduced the existing metals by 84.2% (Cd (II)), 48.8% (Fe (II)), 84.8% (Pb (II)), 52.6% (Cr (VI)), and 52.6% (Mn (II)). These values can be enhanced by further optimization studies on the retention conditions, especially the bed thickness, or by designing the system to include several beds of Ca-MCM particles.

## Environmental implications

The study involved the health risk assessments of heavy metal contamination in groundwater in Siwa Oasis and their potential environmental decontamination using safe and effective Ca-MCM-41 adsorbent. Results showed that Fe, Cd, Cr, Pb, and Mn exceeded WHO guidelines. Non-carcinogenic risks were significant, particularly for Cd, Cr, and Pb through ingestion based on the hazard index (HI) values. Carcinogenic risks from Cd, Cr, and Pb were also high. Calcium-rich MCM-41 obtained using natural marble showed significant performances in the removal of most of these toxic ions.

## Supplementary Information

Below is the link to the electronic supplementary material.

Supplementary Material 1

## Data Availability

The data will be available up on request to corresponding author.
