# Supplementary material for: Calcium modified mesoporous silica from marble for the removal of cadmium, lead, chromium, iron, and manganese from Siwa Oasis groundwater
Source: Sci Rep. 2025 Aug 25;15:31299. doi: 10.1038/s41598-025-15802-2 (PMC12379000; doi:10.1038/s41598-025-15802-2)
Supplement: Supplementary file 1 — Supplementary Material 1 [file 41598_2025_15802_MOESM1_ESM.docx]

**Calcium modified mesoporous silica from marble for the removal of cadmium, lead, chromium, iron, and manganese from Siwa Oasis groundwater**

**Mohamed Hamdy Eid ^1,2^, Attila Kovács^1^, Péter Szűcs^1^, Mohamed Shaban ^3*^, A. M. Elbasiony^4^, Ahmed Mehaney ^5^, Haifa A. Alqhtani^6^, Ahmed A. Allam^7^, Mostafa R. Abukhadra٭^8, 9^**

^1^Institute of Environmental Management, Faculty of Earth Science, University of Miskolc, 3515 Miskolc- Egyetemváros, Hungary

^2^Geology Department, Faculty of Science, Beni-Suef University, Beni-Suef, 65211, Egypt

^3^Department of Physics, Faculty of Science, Islamic University of Madinah, P. O. Box: 170, Madinah 42351, Saudi Arabia

^4^Department of Chemistry, College of Science, Northern Border University (NBU), Arar, Saudi Arabia

^5^Physics Department, Faculty of Science, Beni-Suef University, Beni Suef 62512, Egypt

^6^Department of Biology, college of Science, Princess Nourah bint Abdulrahman University, P.O. BOX 84428, Riyadh 11671, Saudi Arabia

^7^Department of Biology, College of Science, Imam Mohammad Ibn Saud Islamic University, Riyadh 11623, Saudi Arabia

^8^Geosciences Department, College of Science, United Arab Emirates University, 15551, Al Ain, United Arab Emirates.

^9^Applied Science Research Center, Applied Science Private University, Amman, Jordan

Corresponding author٭: [Abukhadra89@Science.bsu.edu.eg](mailto:Abukhadra89@Science.bsu.edu.eg) (M.R. A.)

**Table. S1.** The standard values used for risk evaluation [1]

| HM | Cd | Cr | Cu | Fe | Mn | Ni | Pb | Zn |
| --- | --- | --- | --- | --- | --- | --- | --- | --- |
| **RfD Oral(mg/kg/day)** | **0.0005** | **0.003** | **0.04** | **0.7** | **0.024** | **0.02** | **0.0014** | **0.3** |
| **ABS** | **0.05** | **0.025** | **0.3** | **0.2** | **0.04** | **0.04** | **0.3** | **0.2** |
| **Rfd Dermal (mg/kg/day)** | **0.000025** | **0.000075** | **0.012** | **0.14** | **0.00096** | **0.0008** | **0.00042** | **0.06** |
| **CSF oral mg/kg/day** | **6.1** | **0.5** |  |  |  |  | **0.5** |  |
| **CSF dermal** | **6100** | **500** |  |  |  |  | **500** |  |
| **Kp** | **0.001** | **0.002** | **0.001** | **0.001** | **0.001** | **0.0002** | **0.0001** | **0.0006** |
| **Si** | **0.003** | **0.05** | **3** | **0.3** | **0.05** | **0.07** | **0.01** | **1** |
| **ET Adult (h/day)** | **0.58** | | | | | | | |
| **ET Child (h/day)** | **1** | | | | | | | |
| **SA Adult (cm^2^)** | **18000** | | | | | | | |
| **SA Child (cm^2^)** | **6600** | | | | | | | |
| **CF (L/cm^3^)** | **0.001** | | | | | | | |
| **IR Adult (L/day)** | **2.2** | | | | | | | |
| **IR Child (L/day)** | **1.8** | | | | | | | |
| **EF (day/year)** | **350** | | | | | | | |
| **ED Adult (year)** | **70** | | | | | | | |
| **ED Child (year)** | **6** | | | | | | | |
| **BW Adult (kg)** | **70** | | | | | | | |
| **BW Child (kg)** | **15** | | | | | | | |
| **AT Adult (day)** | **25550** | | | | | | | |
| **AT Child (day)** | **2190** | | | | | | | |

**Table S2.** Nonlinear equations of kinetic, classic isotherm, and advanced isotherm models [2, 3]

| Kinetic models | | |
| --- | --- | --- |
| Model | **Equation** | **Parameters** |
| Pseudo-first-order | $Q_{t}=Q_{e} (1-e^{{-k}_{1}.t})$ | Q_t_ (mg/g) is the adsorbed ions at time (t), and K_1_ is the rate constant of the first-order adsorption (1/min) |
| Pseudo-second-order | $Q_{t}=\frac{Q_{e}^{2}k_{2}t}{1+Q_{e}k_{2}t}$ | Qe is the quantity of adsorbed ions after equilibration (mg/g), and K_2_ is the model rate constant (g/mg min). |
| Classic Isotherm models | | |
| Model | **Equation** | **Parameters** |
| Langmuir | $Q_{e}=\frac{Q_{max} bC_{e}}{(1+bC_{e})}$ | *C_e_* is the rest ions concentrations (mg/L), *Q_max_* is the theoritical maximum adsorption capacity (mg/g), and *b* is the Langmuir constant (L/mg) |
| Freundlich | $Q_{e}=K_{f}C_{e}^{1/n}$ | K_F_ (mg/g) is the constant of Freundlich model related to the adsorption capacity and n is the constant of Freundlich model related to the adsorption intensities |
| Dubinin–Radushkevich | $Q_{e}=Q_{m}e^{-\betaɛ^{2}}$ | β (mol^2^/KJ^2^) is the D-R constant, ɛ (KJ^2^/mol^2^) is the polanyil potential, and Q_m_ is the adsorption capacity (mg/g) |
| Advanced isotherm models | | |
| Model | **Equation** | **Parameters** |
| Monolayer model with one energy site (Model 1) | $Q=nN_{o} =\frac{nN_{M}}{1+{(\frac{C1/2}{C})}^{n}}=\frac{Q_{o}}{1+{(\frac{C1/2}{C})}^{n}}$ | Q is the adsorbed quantities in mg/g  n is the number of adsorbed ion per site  Nm is the density of the effective receptor sites (mg/g)  Q_o_ is the adsorption capacity at the saturation state in mg/g  C1/2 is the concentration of the ions at half saturation stage in mg/L  C1 and C2 are the concentrations of the ions at the half saturation stage for the first active sites and the second active sites, respectively  n1 and n2 are the adsorbed ions per site for the first active sites and the second active sites, respectively |
| Monolayer model with two energy sites (Model 2) | $Q=\frac{n_{1}N_{1M}}{1+{(\frac{C_{1}}{C})}^{n_{1}}}+\frac{n_{2}N_{2M}}{1+{(\frac{C_{2}}{C})}^{n_{2}}}$ |  |
| Double layer model with one energy site (Model 3) | $Q=Q_{o}\frac{({\frac{C}{C1/2})}^{n}+2({\frac{C}{C1/2})}^{2n}}{1+({\frac{C}{C1/2})}^{n}+({\frac{C}{C1/2})}^{2n}}$ |  |
| Double layer model with two energy sites (Model 3) | $Q=Q_{o}\frac{({\frac{C}{C1})}^{n}+2({\frac{C}{C2})}^{2n}}{1+({\frac{C}{C1})}^{n}+({\frac{C}{C2})}^{2n}}$ |  |

**References:**

1. Eid, M.H., Eissa, M., Mohamed, E.A. et al. New approach into human health risk assessment associated with heavy metals in surface water and groundwater using Monte Carlo Method. Sci Rep **14**, 1008 (2024). https://doi.org/10.1038/s41598-023-50000-y.
2. X. Yang, J. Wang, A.M. El-Sherbeeny, A.A. AlHammadi, W.-H. Park, M.R. Abukhadra, Insight into the adsorption and oxidation activity of a ZnO/piezoelectric quartz core-shell for enhanced decontamination of ibuprofen: steric, energetic, and oxidation studies, Chemical Engineering Journal 431 (2022) 134312
3. A.M. Ahmed, I. Saad, M.A. Rafea, M.R. Abukhadra, Synergetic and advanced isotherm investigation for the enhancement influence of zeolitization and β-cyclodextrin hybridization on the retention efficiency of U(vi) ions by diatomite, RSC Advances 14 (2024) 8752–8768.
